# Quantum Chess as a Pedagogical Tool for Teaching Quantum Information Science in High Schools

**DOI:** 10.1021/acs.jchemed.5c00836

**Published:** 2026-06-08

**Authors:** Padmanabh Kaushik, Nam P. Vu, Crystal Yeung, Swetha Tadisina, Leah Boyle, Vedit Venkatesh, Maya Zilberstein, Nicholas Sorak, Kusum Subedi, Delmar G. A. Cabral, Brandon Allen, Victor S. Batista, Heidi P. Hendrickson

**Affiliations:** † Department of Chemistry, 3091 Lafayette College, Easton, Pennsylvania 18042, United States; ‡ Department of Biomedical Engineering, Faculty of Engineering and Information Technology, University of Melbourne, Victoria 3010, Australia; ¶ Department of Electrical Engineering and Computer Science, 2167 Massachusetts Institute of Technology, Cambridge, Massachusetts 02139, United States; § Department of Chemistry, 5755 Yale University, New Haven, Connecticut 06520, United States; ∥ Yale Quantum Institute, 5755 Yale University, New Haven, Connecticut 06511, United States

**Keywords:** Quantum Information Science & Engineering, QISE, High School, Quantum Chess, Quantum Games, Quantum Mechanics, Superposition, Introductory Chemistry

## Abstract

Recent advances in quantum information science and engineering (QISE)—spanning theoretical algorithms and quantum hardware development—have intensified the need for broader education in foundational quantum concepts. Yet, the abstract and counterintuitive nature of quantum phenomena presents major barriers to incorporating QISE into high school chemistry curricula. To address this, we developed a game-based instructional workshop designed to introduce secondary students to key quantum mechanical principles, such as quantum superposition and measurement. Grounded in experiential learning theory, the workshop combined guided exploration, structured gameplay, and conceptual explanation to foster engagement and understanding. Pre- and postworkshop assessments indicated high levels of student engagement, as well as demonstrated and perceived learning. These results demonstrate the potential of combining game-based approaches with targeted instruction and collaborative activities to create effective tools for introducing QISE at the secondary level.

## Graphical abstract

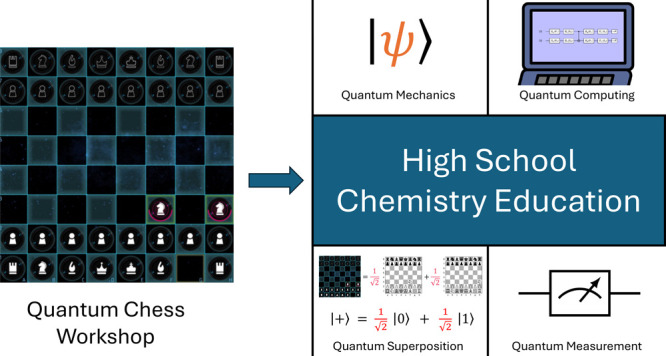

## 1 Introduction

### 1.1 The Rise of Quantum Computing

Quantum computing is increasingly viewed as the next major technological leap following artificial intelligence. Recent recognition of foundational work in superconducting circuits—most notably the 2025 Nobel Prize in Physics awarded to John Clarke, Michel H. Devoret, and John M. Martinis for uncovering macroscopic quantum tunneling and energy quantization in electrical circuits—highlights how early insights into quantum behavior of engineered devices ultimately enabled today’s superconducting quantum computers. Since the first proposals for quantum computation in the 1980s,^1^ the field has moved from abstract theory to specialized quantum algorithms in the 1990s^2–4^ that outperform classical approaches. Experimental progress followed across multiple platforms, including NMR,^5,6^ trapped ions,^7^ photonics,^8^ and superconducting circuits via circuit QED.^9,10^ Together, these advances have established quantum information science and engineering (QISE) as a rapidly expanding discipline with the potential to reshape computation, simulation, and data processing.^11–14^

These advancements raise an important question for educators: how can we prepare the next generation of chemists to contribute meaningfully to QISE?^15^ Many QISE concepts map naturally onto chemical problems: superposition ↔ delocalized electronic/molecular configurations, measurement ↔ experimental observation of specific reaction products, and entanglement ↔ correlated electronic or vibronic states. A challenge lies in enabling aspiring chemists to understand core quantum concepts and their direct applications to spectroscopy, catalysis, and electronic-structure simulation.

Although postsecondary programs are beginning to respond by creating interdisciplinary centers and programs—for example, Yale College’s Certificate in Quantum Science and Engineering—quantum information science is still not taught consistently within the undergraduate chemistry curriculum. For example, in 2022 the Lowering Activation Barriers to Success in Physical Chemistry (LABSIP) group asked approximately 170 physical chemistry instructors to rank multiple quantum chemistry topics according to importance in a physical chemistry course (with topics taken from the table of contents of a popular physical chemistry textbook).^16^ While the “postulates of quantum mechanics” was ranked second out of approximately 70 topics, the topic of “superpositions” ranked 34th and the topic of “entanglement” was not included in the list of topics, thus illustrating the variability with which instructors may relate these topics to QISE.^16^ Physical Chemistry textbooks that do describe these sorts of foundational QISE concepts (e.g., superposition and entanglement), tend to include those discussions in supplemental chapters, enabling instructors to incorporate QISE as they see fit.^17^ Similar variation is also present at the introductory level. For example, multiple general chemistry texts briefly mention superposition and/or entanglement, often referencing the Schrödinger’s cat thought experiment, though most do not explore these concepts in depth.^18,19^ As a result of the variability in QISE concepts across the undergraduate chemistry curriculum, chemists often have to wait until graduate school to engage with and contribute to QISE.

In general, quantum mechanics education in chemistry tends to focus on applications in molecular systems, such as electronic structure and spectroscopy, without explicitly mapping these to QISE topics. For example, in the LABSIP study, the other topics that made the top five most important were “the Schrödinger equation”, “vibrational energy levels of molecules”, “the quantum mechanical harmonic oscillator”, and “atomic orbitals”.^16^ These sorts of quantum chemistry concepts are covered in depth in upper-level physical chemistry courses, and are also introduced briefly in general chemistry courses. Computational and visualization tools, such as WebMO,^20^ make it increasingly possible to introduce these concepts earlier on in the chemistry curriculum.^21–25^ In the United States, the Next Generation Science Standards (NGSS) for high school explicitly address aspects of atomic and molecular systems that can be supported by such computational tools, e.g., many standards under HS-PS1, HS-PS3, which cover energy levels in atoms and energetics of atomic and molecular interactions. Thus, there has been a significant effort to extend the use of these tools into the high school chemistry curriculum.^26–29^

On the other hand, the NGSS do not explicitly address QISE, though certain standards, such as HS-PS4–2 and HS-PS4–5, provide entry points through topics like digital transmission and wave behavior.^30^ Even in regions where quantum mechanics appears in secondary education, coverage of QISE topics is often fragmented, omits essential ideas, and is based in the physics curriculum.^31^ International comparisons reflect similar inconsistencies; only 2 of 15 countries surveyed in a recent study include quantum entanglement in their high school physics curricula.^32^ Teaching QISE at this level is inherently challenging, as students frequently conflate classical and quantum explanations of physical phenomena, making it difficult to grasp concepts such as superposition, entanglement, and probabilistic measurement.^33^ As a result, an active area of educational research focuses on developing more effective strategies for introducing these core quantum ideas.^32–34^ These efforts have mostly taken place in the context of physics education, rather than in chemistry. However, previous success with using computational tools to integrate quantum chemistry-related concepts in high school education provides a precedent for engaging high school chemistry students with foundational quantum concepts.^26–29^

Game-based learning (GBL) has emerged as a promising approach to this challenge. Quantum-themed games create interactive settings that help make abstract ideas more concrete, promoting conceptual engagement while reducing cognitive load.^35–42^ Despite this potential, most reports on QISE-focused games emphasize the mechanics and design of the games themselves, with far fewer offering concrete examples of how such games can be integrated into high school classrooms.^35^ In this work, we address that gap by presenting a high school–level workshop built around the popular *Quantum Chess* game.^41,43^ The workshop blends gameplay with guided instruction in quantum mechanics, explicitly linking in-game dynamics to core QISE concepts. We also analyze pre- and postworkshop survey data to assess both conceptual learning gains and students’ self-reported understanding following their participation.

### 1.2 Scope of QISE in the US K-12 Education System

As of fall 2023, about 15.6 million students were enrolled in US public high schools.^44^ In Pennsylvania, where the workshop was conducted, public schools enrolled approximately 0.6 million high school students in the 2024−2025 academic year.^45^ This scale highlights both the reach and challenges of including advanced concepts like QISE into traditional high-school curricula. In the United States, K–12 students, on average, attend school for about 6.9 h a day for 179 days per calendar year, which amounts to approximately 1,235 h spent in schools annually.^46^ Within this time, students are required to take subjects such as general mathematics, physics, and chemistry; however, advanced placement electives (AP Calculus, AP Physics, AP Chemistry), which may have the scope to cover QISE-related concepts, remain optional or may not be offered at particular schools. Additionally, the hours spent on STEM education might vary from school to school and state to state, as highlighted by some nationwide surveys. A study by Kolbe et al.^47^ points out that at the eighth-grade level, only about one-third of classrooms provided at least 5 h of science instruction per week, the threshold associated with more inquiry-based teaching practices.

It is challenging for students to develop a conceptual base for QISE without access to advanced physics and chemistry courses where key concepts such as quantum superposition and measurement are taught. Furthermore, many studies indicate systemic inequities related to students’ access to computer science, computational thinking, and coding instruction, which are foundational concepts for quantum computing. Studies by Grover and Pea (2013),^48^ Kwon, Lee, & Kim (2025),^49^ Margolis et al. (2008),^50^ and Weintrop & Wilensky (2015)^51^ highlight how barriers in computational and programming instruction can negatively impact student engagement in computing-intensive fields.

Many existing QISE software packages (such as Qiskit^52^ and PennyLane^53^) and freely available teaching materials are designed for college students and more advanced researchers. These teaching resources assume prior knowledge of mathematics, programming, and other concepts in quantum physics, which can limit their effectiveness in reaching all students at the high school level without additional pedagogical resources. Hence, introducing QISE in high schools does not encompass only the development of new curricula but also structural and pedagogical innovations that will equip teachers and prepare students with necessary foundational skills to meaningfully engage with more advanced resources in quantum computing. Our work seeks to pave a path that enables high school educators to teach quantum mechanics—and, by extension, quantum computing—using popular, low-cost resources while addressing the pedagogical barriers posed by current educational structures and materials.

### 1.3 Game-based Learning in QISE via Quantum Chess

The basis of game-based learning (GBL) strategies is that hands-on experience enhances perception and learning of abstract concepts. Many studies have shown that GBL is effective for experiential learning and active retention of critical knowledge.^54,55^ Educational games present abstract scientific problems as interactive challenges that promote procedural thinking and problem-solving skills by breaking down complex topics into simpler steps, giving instant feedback, and creating a safe learning space without fear of failure.^56–58^ As a result, various games are being used by educators worldwide to help students learn complex concepts in physics and chemistry in a manageable way and through a risk-free learning environment.^56–61^

GBL has shown particular promise in STEM education, where abstract content and traditional didactic methods can limit student engagement and comprehension. Notable examples include *Foldit*,^57^ a puzzle-based game that teaches protein folding, and *Amino-structure*,^62^ a card game that introduces amino acid properties. These tools enhance learning by transforming complex biochemical concepts into interactive challenges. Despite the success of GBL in various scientific disciplines, there remains a lack of empirical studies examining its effectiveness in teaching quantum mechanics—particularly at the high school level.

We suspect that this gap in the literature may be due to the pedagogical challenges of connecting in-class gameplay to the quantum-mechanical concepts demonstrated in QISE games. To bridge this gap, we implement a quantum-game-based workshop that combines multiple interactive resources to teach students about the quantum-mechanics concepts they observe during gameplay. Specifically, we based the workshop on *Quantum Chess*, a popular educational game developed by Quantum Realm Games,^43^ which integrates key principles of quantum mechanics into a modified version of chess. The game introduces players to nonclassical phenomena such as superposition and probabilistic measurement, enabling them to explore these concepts through direct interaction with quantum-inspired game mechanics. A summary of *Quantum Chess* is provided in the Supporting Information, and a complete description of the game can be found in ref 41.

## 2 Activity Description

### 2.1 Prior Work

The workshop was initially piloted in August 2023 at Yale University through the Yale Pathways to Science Program. Approximately 30 students from diverse backgrounds, representing 10 high schools across New Haven County in Connecticut, participated. The workshop was also run at the IEEE Integrated STEM Education Conference in March 2024.^63,64^ Feedback from the pilots enabled the authors to revise the workshop prior to implementing it in a public, Pennsylvania high school.

We were initially inspired to use quantum chess because the chess board configurations and game-play dynamics can be related to molecular configurational dynamics on surfaces or in small clusters. For example, each chess square could be viewed as a site on a catalytic surface and each chess piece as a molecule; a “split” move represents a molecule in a coherent superposition of conformations, and a measurement corresponds to an experimental probe selecting one configuration. Thus, we expected that presenting quantum ideas through a medium that can be mapped to molecular examples (e.g., alternate adsorption sites, reaction pathways, or resonance structures) could lower the barrier for chemistry students to engage with QISE.

### 2.2 Workshop Description

The Quantum Games for Quantum Computing workshop (provided in full in the Supporting Information) was designed as a 90 min after-school workshop and implemented at a Pennsylvania public high school in April 2024. It was designed for participants with no prior experience in QISE. An outline of the workshop structure is provided in Figure 1. Twenty minutes of the workshop was dedicated to pre- and postworkshop surveys. Additional time (5–10 min) was spent on introductions and closing. As such, the 65 min of QISE content in the workshop could, for example, be adapted to a lesson plan for two class sessions in a high school physics or chemistry course. At the start of the workshop, participants were asked to complete a preworkshop survey. To begin the workshop, facilitators introduced themselves and familiarized participants with the schedule. Then, participants watched a short introductory video on quantum computing, qubits, and possible applications or fields of research.^65^ The video was developed by the Yale Quantum Institute and included commentary from Nobel Laureate Michel Devoret and other pioneers in the field.

**Figure 1:**
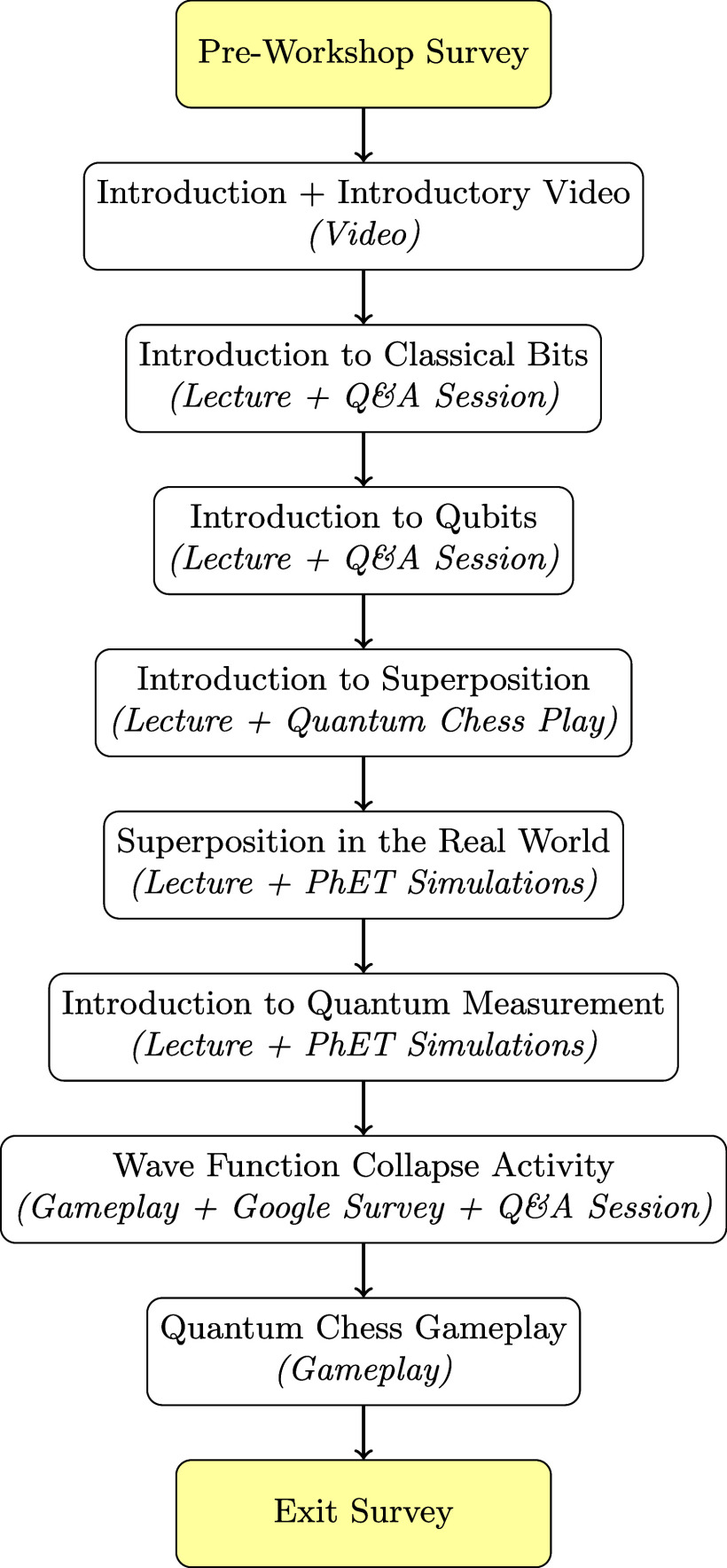
Workshop structure showing the sequence of activities and their instructional components in parentheses.

Phase 1 (Introduction to Classical Computing): The Quantum Games for Quantum Computing workshop began with an introduction to the most fundamental concept in classical computing—the bit. Facilitators introduced the concept of classical bits using the analogy of an unbiased coin, where heads represents 0 (logic low) and tails represents 1 (logic high). Dirac (bra-ket) notation was introduced to describe a classical bit, familiarizing participants with the quantum notation, as shown in Figure 2(a). While Dirac notation is not typically used for classical state notation, the idea was to introduce the new notation in a familiar context so that participants could focus on the new linear algebra concepts being introduced. Although Figure 2(a) is shown as a completed slide, the workshop slides were built up line-by-line to give participants time to understand the progression between equations. Facilitators explained that the state of a classical bit can only be described by one state at any given time, meaning that when the bit is in state |0⟩ (heads), the coefficient for |0⟩ must be 1 while the coefficient for |1⟩ must be 0, and vice versa for the |1⟩ state (tails).

**Figure 2:**
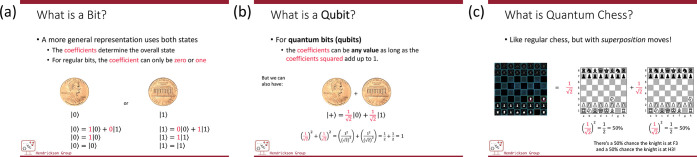
Workshop slides showing the heads/tails representation of (a) a classical bit and (b) a qubit, alongside a Dirac (bra-ket) notation representation for both classical and quantum bits. (c) Workshop slide showing a “split” move as a superposition of two distinct moves with equal coefficients that result in a 50% chance of observing the chess piece in either position. Adapted with permission from Quantum Realm Games. https://quantumrealmgames.com/ [Accessed 03–07–2024].^43^ Copyright 2025 Quantum Realm Games.

Phase 2 (Introduction to Quantum Bits): Once students were familiar with classical bits, the workshop transitioned into the concept of quantum bits (qubits), as shown in Figure 2(b). It was explained to participants that every qubit state could be a superposition of states |0⟩ and |1⟩, with the overall qubit state described mathematically as

|qubit⟩=α|0⟩+β|1⟩ 1

where instead of being restricted to only 0 and 1, the coefficients could take any value as long as

$$\alpha^{2}+\beta^{2}=1$$ 2

For simplicity, and because the workshop considered measurement in the |0⟩/|1⟩ basis, we take α and β to be real; relative phases are omitted. The coefficients were shown to be related to the probability of observing one of the states in the superposition. For example, participants were shown that in the case of an unbiased coin, $\alpha =\beta =\frac{1}{\sqrt{2}}$ since both heads and tails are equally likely.

Phase 3 (Superposition in Quantum Chess): The participants were then introduced to quantum chess. Facilitators explained how superposition could be used in moving different pieces in the chessboard. For those unfamiliar with classical chess, a packet summarizing key chess concepts was provided (available in Supporting Information). The instructors explained how in Quantum Chess, compared to traditional chess, a single move can place a piece in a superposition of multiple positions at once. For instance, as demonstrated in Figure 2(c), participants were first shown an example of how a knight moves in classical chess—such as moving from square g1 to f3 or h3—but existing in only one of those positions at a time. In Quantum Chess, however, the same knight can perform a split move, entering a superposition of both possible positions simultaneously. This means the knight can exist at f3 and h3 at the same time. The superposition move in quantum chess was related back to qubit superposition by using chess boards to represent the possible system states instead of Dirac notation, with the same color-coded coefficients representing a 50% chance of observing each state. Facilitators then walked participants through the brief hands-on tutorial provided as part of the *Quantum Chess* game, and participants were given time to explore the quantum chess game on their own, while facilitators walked around the room to interact with and guide the participants.

Phase 4 (Introduction to Superposition in the Real World): To connect quantum mechanics concepts to their demonstration in the quantum chess game, the workshop incorporated Physics Education Technology (PhET) simulations from the University of Colorado Boulder.^66,67^ Facilitators used the Wave Interference^66^ and Quantum Wave Interference^67^ demonstrations to explain the physics behind quantum superposition and wave-particle duality, and participants were encouraged to follow along using the demonstration. As shown in Figure 3(a), two light waves can create a superposition wave that creates an interference pattern on a detector screen, and a double-slit apparatus produces an interference pattern analogous to that from two coherent sources.^66^ Quantum superposition was then explained in the context of the double-slit experiment, using the Quantum Wave Interference demonstration (Figure 3(b,c)), where individual photons or electrons were fired from a source, one at a time, through a double-slit apparatus.^67^ Facilitators described the interference pattern observed for quantum particles as the result of a superposition wave function with a 50% chance of the quantum particle traveling through either slit 1 or slit 2. Mathematically, participants were shown the Dirac notation representation of the superposition and connected it to other superpositions described in the workshop.

**Figure 3:**
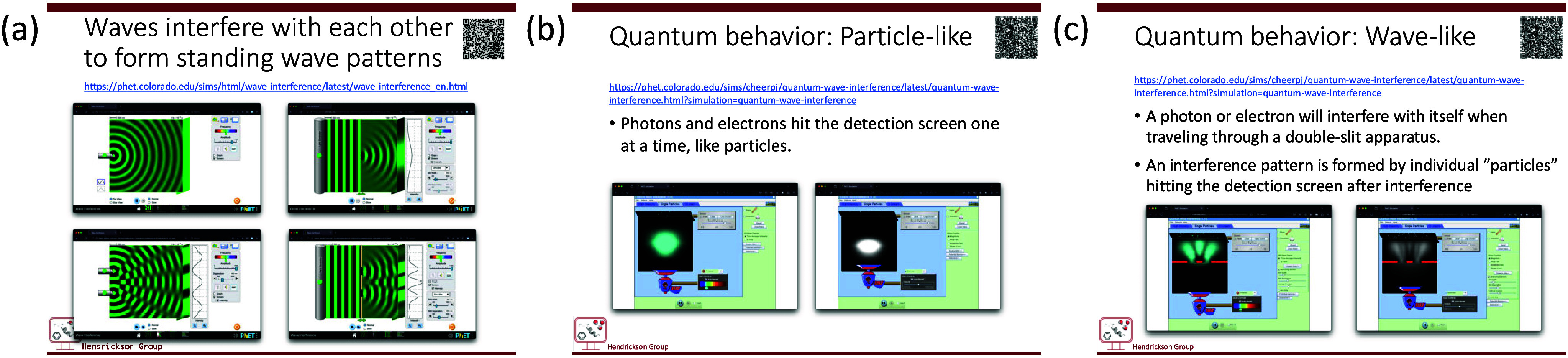
Workshop slide utilizing (a) the Wave Interference PhET demonstration^66^ to show the interference patterns produced by the superposition of two light waves, and the Quantum Wave Interference PhET demonstration^67^ to show (b) the particle-like behavior of quantum particles, and (c) the wave-like behavior of quantum particles that results in an interference pattern.

Phase 5 (Introduction to Quantum Measurement): At this point, the workshop progressed to discuss the concept of quantum measurement. Facilitators explained that measuring a quantum state causes it to collapse from a superposition into one of the states that make up the superposition. This process, known as wave function collapse, is inherently probabilistic rather than deterministic. The workshop used the double-slit experiment to demonstrate this effect: when no detector measures which slit the electron passes through, an interference pattern emerges, indicating superposition. However, once a measurement is made to determine which slit the electron goes through, the interference pattern disappears and the pattern on the screen is as if the particles were going through one slit at a time. This transition from uncertainty to definiteness upon measurement mirrored how a qubit’s probabilistic wave function becomes a definite bit of information once measured.

Phase 6 (Wave Function Collapse Activity): Following the scientific explanation, participants engaged in a hands-on activity demonstrating quantum probabilistic measurement outcomes. In *Quantum Chess Puzzle* 2, a knight performs a split move (to B5 and E2) to capture the rook at G1, as shown in Figure 4(a). The knight has an equal probability of being at B5 or E2, but successful capture of the rook is only possible if the knight is at E2, meaning that capture is not guaranteed. Participants attempted to solve the activity on their own for six different trials and recorded how many times the capture succeeded via a Google Form (provided in the Supporting Information).

**Figure 4:**
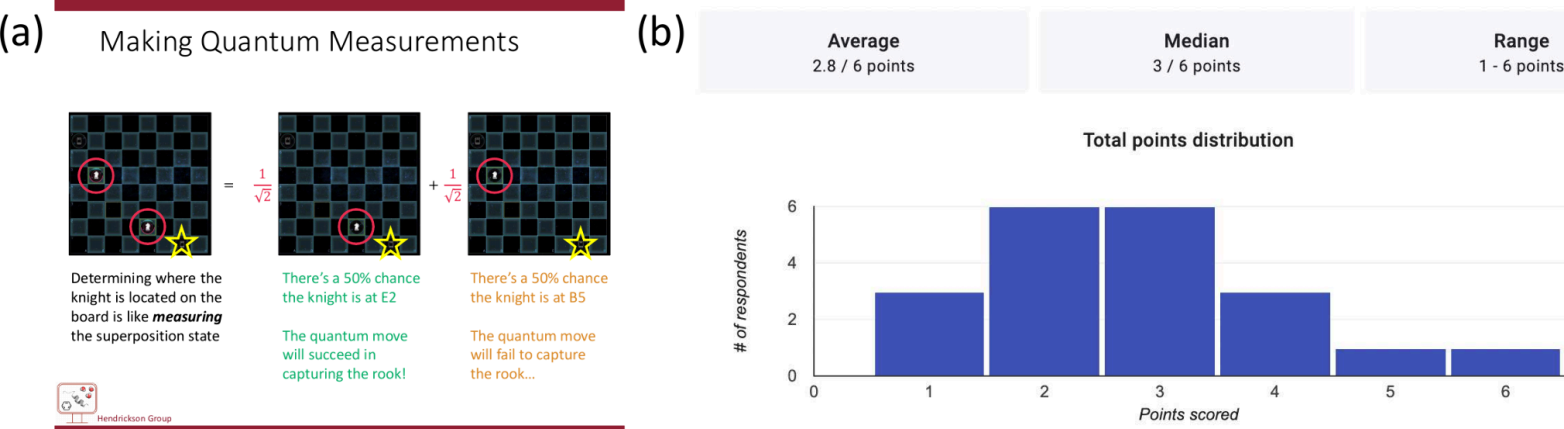
(a) Workshop slide illustrating probabilistic measurement outcomes in the context of the quantum chess puzzle. Adapted with permission from Quantum Realm Games. https://quantumrealmgames.com/ [Accessed 03–07–2024].^43^ Copyright 2025 Quantum Realm Games. (b) Participant reported number of successful captures out of six trials as collected and visualized (in real-time during the workshop) using a Google Form. (Twenty responses are shown because participants were able to submit the form multiple times.)

The results of the activity, shown in Figure 4(b), were shared with the participants in real-time. The expected mean is 3, while the value observed for the workshop participants is 2.8, and this small difference, highlighted by facilitators, provoked further group discussion of probability and quantum measurement.

Finally, participants were provided additional time to complete the trials and explore the quantum chess game again, with facilitator guidance. Once participants had finished using Quantum Chess, the workshop concluded with a general summary and a review of QISE applications.

### 2.3 Data Collection and Evaluation

Workshop participants were high school students from a public high school in Pennsylvania. This study protocol was approved by the Institutional Review Board of Lafayette College Proposal No. AY2324–50. No unexpected or unusually high safety hazards were encountered. All 19 participants and their legal guardians provided informed consent to participate in this study. Participants were asked to complete a preworkshop survey, to determine their initial level of understanding of quantum mechanics concepts, and a postworkshop survey to assess how their level of understanding changed as a result of the workshop. Both the pre- and postworkshop surveys are provided in the SI.

Sixteen participants completed the preworkshop survey. Thirteen participants answered most of the postworkshop questions, with 10 completing the entire survey. Of the 16 participants who completed the preworkshop survey, 19% were in ninth grade (3/16), 19% in 10th grade (3/16), 31% in 11th grade (5/16), and 31% in 12th grade (5/16). Almost 94% (15/16) of students planned to attend college in the future. On average, participants said they were interested in science, quantum mechanics, computer games, and other topics related to the workshop, though they all reported having little to no prior knowledge of quantum mechanics or quantum computing (data are provided in the Supporting Information).

The sample size in this study is too small to draw general conclusions about the impact of the workshop on students’ understanding of quantum mechanical concepts. However, we did proceed to evaluate the results of the surveys to provide a starting point for examining how these sorts of workshops can influence student conceptual understanding in the future. Participants were asked to define wave-particle duality, wave function collapse, quantum superposition, and quantum measurement in both the pre- and postworkshop surveys. Ten participants completed both pre- and postworkshop surveys, and their responses were coded by 11 workshop organizers, including undergraduate students, graduate students, and a professor. Coders utilized a three-point scale ranging from −1 to 1, where −1 meant that the participant’s explanation of a concept became worse after the workshop, 0 meant that their understanding remained the same, and 1 meant their understanding improved.

Improvement was classified as a more accurate description of the quantum concept than the initial description. For example, an improved response for superposition was “Position in space and time” in the preworkshop presurvey, and “A particle having a 50/50 chance to be in one position or another” in the postworkshop survey. Some answers provided in the postworkshop survey were recorded as improved because the participant did not know about the concept, or had an incorrect conception, even though the answer may not have been 100% correct. For example, an improved response for wave function collapse was “idk” (meaning, “I don’t know”) in the preworkshop survey, and “When measured a quantum particle acts like a particle and not a wave” in the postworkshop survey. If greater than 50% of the coders agreed a participant’s understanding improved, the explanation was recorded as an “improved” understanding. Otherwise, the explanation was recorded as “remained the same or became worse.”

In the postworkshop survey, participants were also asked questions on their perceptions of how the workshop helped them improve their understanding of quantum mechanics concepts as well as whether they enjoyed the activities.

## 3 Results

### 3.1 Impact on Participant Understanding

Participants were asked to define wave-particle duality, wave function collapse, quantum superposition, and quantum measurement in both the pre- and postworkshop surveys. The results of comparing participant pre- and post-test responses are provided in Figure 5. Overall, understanding of quantum mechanics concepts improved after participating in the workshop, however, the impact varied by topic. Understanding of wave-particle duality increased for six respondents, understanding of superposition increased for eight, understanding of wave function collapse increased for five, and understanding of quantum measurement increased for six respondents. We suspect that the greatest increase in participant understanding occurred for superposition because this topic was reiterated in multiple contexts throughout the workshop. On the other hand, wave function collapse was only discussed just prior to the measurement activity.

**Figure 5:**
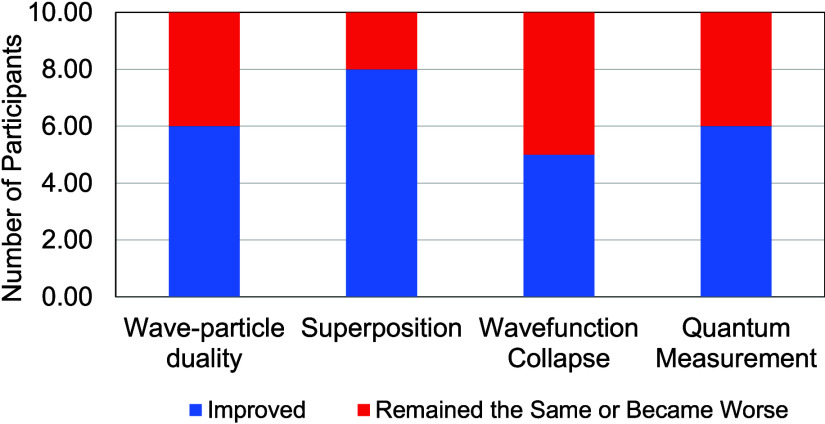
Number of respondents with an understanding of each topic that improved (blue) or that remained the same or became worse (red), as evidenced by participant responses to pre- and postworkshop surveys.

### 3.2 Impact on Participant Perceptions of Understanding

Participants were also asked about their perceptions of how the workshop impacted their understanding of the quantum superposition and quantum measurement. Only 13 participants responded to these questions, so while the conclusions drawn from the data may not be generalizable, they do provide insight for educators to consider when implementing these activities. As shown in Figure 6, when asked about improvement in their understanding of quantum superposition, six respondents expressed strong agreement, five agreed, one somewhat agreed, and one strongly disagreed. When asked about improvement in their understanding of quantum measurement, five respondents strongly agreed, five agreed, one somewhat agreed, one was neutral, and one strongly disagreed. The participant perceptions mirror the qualitative analysis of their actual learning outcomes, with increased understanding for both quantum superposition and measurement concepts and greater improvement for superposition than measurement. Overall, between 75 and 85% of respondents agreed or strongly agreed that the workshop helped them acquire knowledge about quantum superposition and measurement.

**Figure 6:**
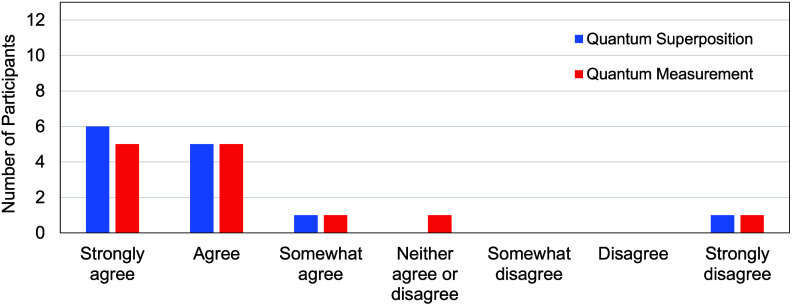
Number of responses to the questions “I know more about quantum superposition after completing the Quantum Chess workshop” (blue) and “I know more about quantum measurement after completing the Quantum Chess workshop” (red).

Finally, to understand student engagement, the postworkshop survey asked participants whether they enjoyed playing the *Quantum Chess* game and solving the *Quantum Chess* puzzles. A total of 13 participants responded, and as shown in Figure 7, all responses were positive. Approximately 85% of respondents indicated they strongly agreed that they enjoyed playing *Quantum Chess*, and 77% strongly agreed that they enjoyed solving the quantum chess puzzles.

**Figure 7:**
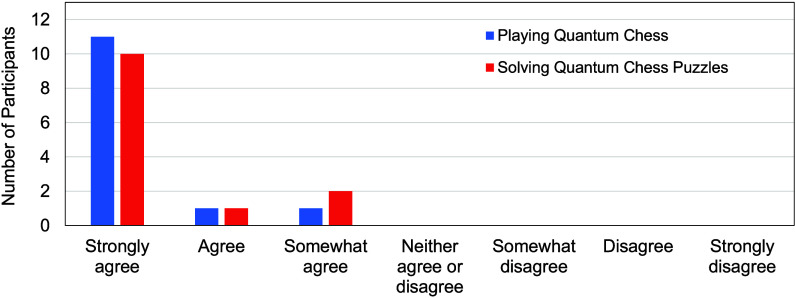
Number of responses to the questions “I enjoyed playing the quantum chess game” (blue) and “I enjoyed solving puzzles using the Quantum Chess Game” (red).

## 4 Conclusions

In the Quantum Games for Quantum Computing workshop, we engaged students from a public high school in Pennsylvania in a 90 min, after-school session designed to introduce core concepts in quantum mechanics and Quantum Information Science and Engineering (QISE). Participants first completed a preworkshop survey to assess their baseline understanding of quantum phenomena. The session began with a lecture-style presentation covering fundamental quantum concepts and their relevance to QISE. Facilitators then guided students through a hands-on tutorial using the *Quantum Chess* game, followed by time for independent exploration. This was followed by a group discussion focused on superposition and probabilistic measurement outcomes. To reinforce these ideas, students completed a collaborative, puzzle-based activity centered on quantum measurement. Finally, a postworkshop survey captured both the participants’ learning gains and their perceptions of what they had learned.

Although the participant sample size was quite small, based on student responses to pre- and postworkshop surveys, the workshop did increase participant understanding (and perception of understanding) of key quantum concepts, particularly the notion of superposition states. Learning outcomes were greater for quantum superposition concepts than quantum measurement concepts, possibly because superposition was reiterated throughout the workshop, while measurement was only discussed toward the end. Future iterations of the workshop could benefit from facilitators reinforcing key QISE concepts during interactive segments—particularly by drawing clearer connections between the measurement activity and quantum computing principles. All participants reported enjoying both the gameplay and the puzzle challenges available within the *Quantum Chess* game. Overall, we conclude that the workshop delivered an engaging and educational introduction to QISE for high school students.

While the survey results provide valuable insights, they are subject to several limitations. The 90 min time frame allowed for only a brief introduction to QISE concepts, limiting the depth and breadth of exposure to the field. Furthermore, the number of workshop participants (19) and survey respondents (10–13, depending on the analysis) was small, which constrains the generalizability of the findings. Participation may have been limited by the after-school format, which required students to opt in and secure parental consent. Implementing the workshop during regular school hours—such as within existing chemistry or physics classes—could help increase accessibility and participation.

Although we implemented a workshop with an interdisciplinary focus, it is also possible to frame this sort of workshop specifically within a chemistry context. Chemists routinely reason about multisite adsorption, resonance, and combinatorial conformational spaces. Thus, framing the quantum concepts in terms of molecular/catalytic analogies, in line with our initial inspiration for using *Quantum Chess*, offers an alternative approach we hope could accelerate transfer from classroom intuition to practical QISE tasks such as variational algorithms for molecular energies or sampling configuration spaces. For instance, a split-knight move could be described to students as a molecule in a coherent superposition of two conformational states, collapsing to one conformation upon measurement (detection or reaction). Then the observed distribution of successful captures can be used as an analogy for product distributions from competing reaction channels when initial states are coherently delocalized across pathways. As an alternate interactive puzzle, students could simulate a molecule with two reaction pathways (A vs B), where a “split” move corresponds to coherent pathway superposition; repeated trials measure product distribution and show the probabilistic outcome of measurement. This is just one example of how the workshop presented here can inspire incorporation of QISE concepts into the introductory chemistry curriculum.

Overall, the results of this study underscore the potential of QISE games combined with targeted instruction to make complex quantum-mechanical concepts more accessible and intuitive for high school students. These findings can inform the design of future educational interventions aimed at introducing quantum science earlier into the academic pipeline. We are optimistic that exposing students to quantum concepts during secondary education will spark sustained interest, encouraging them to pursue further study in QISE and helping to cultivate a well-informed, motivated next generation of quantum professionals.

## Supplementary Material









## Notes

Description of *Quantum Chess* game, pre- and postworkshop surveys; measurement activity Google form, survey responses (PDF)
Traditional chess rules handout (PDF)
Quantum Games for Quantum Computing workshop slides (PDF)
Instructor guide for Quantum Games for Quantum Computing workshop (PDF)
