# Supplementary material for: Quantum Chess as a Pedagogical Tool for Teaching Quantum Information Science in High Schools
Source: J Chem Educ. 2026 Jun 8;103(7):3971–80. doi: 10.1021/acs.jchemed.5c00836 (PMC13374102; doi:10.1021/acs.jchemed.5c00836)
Supplement: Supplementary file 1 [file ed5c00836_si_001.pdf]

Quantum Chess as a Pedagogical Tool for Teaching Quantum Information Science in High Schools

Padmanabh Kaushik,[†,‡] Nam P. Vu,[†,¶] Crystal Yeung,[†] Swetha Tadisina,[†] Leah Boyle,[†] Vedit Venkatesh,[†] Maya Zilberstein,[†] Nicholas Sorak,[†] Kusum Subedi,[†] Delmar G. A. Cabral,[§] Brandon Allen,[§] Victor S. Batista,[*,§,‖] and Heidi P. Hendrickson[*,†]

†*Department of Chemistry, Lafayette College, Easton, PA 18042, USA*

‡*Department of Biomedical Engineering, Faculty of Engineering and Information Technology, University of Melbourne, Victoria 3010, Australia*

¶*Department of Electrical Engineering and Computer Science, Massachusetts Institute of Technology, Cambridge, MA 02139, USA*

§*Department of Chemistry, Yale University, New Haven, CT 06520, USA*

‖*Yale Quantum Institute, Yale University, New Haven, CT 06511, USA*

E-mail: victor.batista@yale.edu; hendrihe@lafayette.edu

1 Superposition and Measurement in Quantum Chess

Here we provide a brief description of the *Quantum Chess* game.[1,2] Generally, each square on a classical chessboard has 2 possible states, just like a classical bit: occupied (1) or unoccupied (0). However, unlike classical bits, quantum chess squares are represented by quantum bits (qubits) as occupied $|1\rangle$ or unoccupied $|0\rangle$. The quantum nature of the qubit allows for superposition of the states in quantum chess. A 64-square chessboard therefore represents a 64-qubit quantum state, which can be represented as the superposition of classical chessboard states,[2]

$$|\psi\rangle = \sum_i A_i |q_0^{(i)}, ..., q_{63}^{(i)}\rangle \tag{1}$$

where A_i is a complex coefficient and $q_j^{(i)} \in \{0 = \text{empty}, 1 = \text{occupied}\}$ and j goes from 0 to 63. For each chess move, the quantum state undergoes a unitary transformation U. Thus, if $|\psi\rangle$ represents the initial chessboard state, and $|\psi'\rangle$ represents the final state, then:

$$|\psi'\rangle = U|\psi\rangle \tag{2}$$

Unlike classical chess, a single piece can occupy multiple squares simultaneously. This phenomenon is best illustrated through the ”split move”, where a piece is put in superposition; it can be moved to two different squares simultaneously. For instance, for a three-qubit system $|001\rangle$, which is a subset of the 64-qubit representation of the chess board, a split move will result in the equally probable states $|010\rangle$ and $|100\rangle$, as described through the following equation:

$$U_{\text{split}}\,|001\rangle = \frac{i}{\sqrt{2}}\left(|010\rangle + |100\rangle\right) \tag{3}$$

To capture the opponent's piece in quantum chess, a quantum measurement is required. A quantum measurement is the physical manipulation of the system of qubits to yield a numerical value. Once the quantum system $|\psi\rangle$ is measured, it collapses into one of the

eigenstates $|a_i\rangle$ with probability $|\langle a_i|\psi\rangle|^2$ (Born's rule). For a quantum system consisting of two binary states, $|\psi\rangle = \alpha|0\rangle + \beta|1\rangle$, and because the measurement operators are Hermitian,[2,3] the probability of the initial state collapsing to state $|0\rangle$ is given by

$$P(0) = |\langle 0| (\alpha |0\rangle + \beta |1\rangle)|^2 = |\alpha \langle 0|0\rangle + \beta \langle 0|1\rangle|^2 = |\alpha|^2 \tag{4}$$

and similarly, $P(1) = |\beta|^2$. Measurement and superposition were explained during the workshop at a simplified level, as described in the Activity Description section.

2 Preworkshop Survey Questions

This survey was provided to participants via the Qualtrics software.

1. First Name
2. Last Name
3. Which high school do you currently attend?
4. What grade are you currently in?
   (a) 9th grade
   (b) 10th grade
   (c) 11th grade
   (d) 12th grade
5. Which high school science classes have you completed so far? Please check all that apply.
   (a) Biology
   (b) Chemistry

(c) Computer Science

(d) Earth Science/Geology

(e) Environmental Science

(f) Psychology

(g) Physics

(h) Other

6. Which high school science classes are you currently enrolled in? Please check all that apply.

   (a) Biology

   (b) Chemistry

   (c) Computer Science

   (d) Earth Science/Geology

   (e) Environmental Science

   (f) Psychology

   (g) Physics

   (h) Other

   The next eight questions used the following Likert scale:

   (a) Not at all interested

   (b) Somewhat interested

   (c) Interested

   (d) Very interested

7. How interested are you in science?

8. How interested are you in chemistry?
9. How interested are you in physics?
10. How interested are you in computing?
11. How interested are you in computer games?
12. How interested are you in puzzle games?
13. How interested are you in quantum mechanics?
14. How interested are you in quantum computing?

    The next two questions used the following Likert scale:

    (a) I know nothing about it

    (b) I know a little about it

    (c) I know a lot about it

15. How much do you know about Quantum Mechanics?
16. How much do you know about Quantum Computing?
17. Do you plan to attend college?

    (a) Yes

    (b) No

    (c) Not sure

    If answered "Yes" to "Do you plan to attend college?"

18. At this moment in time, what field do you intended to major in?

    If answered "No" or "Not sure" to "Do you plan to attend college?"

19. At this moment in time, what type of training or employment do you plan to seek after high school?

    For the next four questions, participations were directed: Please respond to the following prompts to the best of your ability:

20. Describe what the term ”wave-particle duality” means.
21. Describe what the term ”superposition” means.
22. Describe what the term ”wavefunction collapse” means.
23. Describe what the term ”quantum measurement” means.

3 Postworkshop Survey Questions

This survey was provided to participants via the Qualtrics software.

1. First Name
2. Last Name

    The next six questions used the following Likert scale:

    (a) Strongly disagree
    (b) Disagree
    (c) Somewhat disagree
    (d) Neither agree or disagree
    (e) Somewhat agree
    (f) Agree
    (g) Strongly agree

3. I learned a lot about quantum mechanics during the Quantum Chess workshop

4. I learned a lot about quantum computing during the Quantum Chess workshop
5. I enjoyed solving puzzles using the Quantum Chess Game
6. I enjoyed playing the Quantum Chess Game
7. I know more about quantum superposition after completing the Quantum Chess workshop
8. I know more about quantum measurement after completing the Quantum Chess workshop
9. What segment of the workshop did you find to be most interesting?
   (a) Introduction to quantum mechanics and quantum computing
   (b) Initial exploration of Quantum Chess Game
   (c) Explanation of quantum mechanical superposition and measurement
   (d) Group activity to predict measurement outcomes
   (e) Final exploration of Quantum Chess Game
10. What did you find most interesting during the segment of the workshop you selected in Question 9?
11. What segment of the workshop did you find to be the most challenging?
   (a) Introduction to quantum mechanics and quantum computing
   (b) Initial exploration of Quantum Chess Game
   (c) Explanation of quantum mechanical superposition and measurement
   (d) Group activity to predict measurement outcomes
   (e) Final exploration of Quantum Chess Game

12. What did you find most challenging during the segment of the workshop you selected in Question 11?

13. In which segment of the workshop did you learn the most about quantum superposition?

    (a) Introduction to quantum mechanics and quantum computing

    (b) Initial exploration of Quantum Chess Game

    (c) Explanation of quantum mechanical superposition and measurement

    (d) Group activity to predict measurement outcomes

    (e) Final exploration of Quantum Chess Game

14. What helped you learn about quantum superposition during the segment of the workshop you selected in Question 13?

15. In which segment of the workshop did you learn the most about quantum measurement?

    (a) Introduction to quantum mechanics and quantum computing

    (b) Initial exploration of Quantum Chess Game

    (c) Explanation of quantum mechanical superposition and measurement

    (d) Group activity to predict measurement outcomes

    (e) Final exploration of Quantum Chess Game

16. What helped you learn about quantum measurement during the segment of the workshop you selected in Question 15?

17. Do you plan to pursue other quantum games after participating in this workshop?

    (a) Yes

    (b) No

For the next four questions, participations were directed: Please respond to the following prompts to the best of your ability:

18. Describe what the term ”wave-particle duality” means.

19. Describe what the term ”superposition” means.

20. Describe what the term ”wavefunction collapse” means.

21. Describe what the term ”quantum measurement” means.

4 Measurement Activity Google Form

During the measurement activity, participants were provided with a QR code for a Google Form where they could record the number of successful vs unsuccessful trials when solving *Quantum Chess Puzzle* 2. Participants were asked to only record trials where they solved the puzzle correctly.

5 Participant interests and prior knowledge

Sixteen participants responded to the pre-workshop survey. They answered questions about their interests using the following Likert scale, which we converted to a numerical value:

- 0 = Not at all interested
- 1 = Somewhat interested
- 2 = Interested
- 3 = Very interested

As shown in Figure 2, on average, participants were interested in the topics related to the workshop, including quantum mechanics and quantum computing.

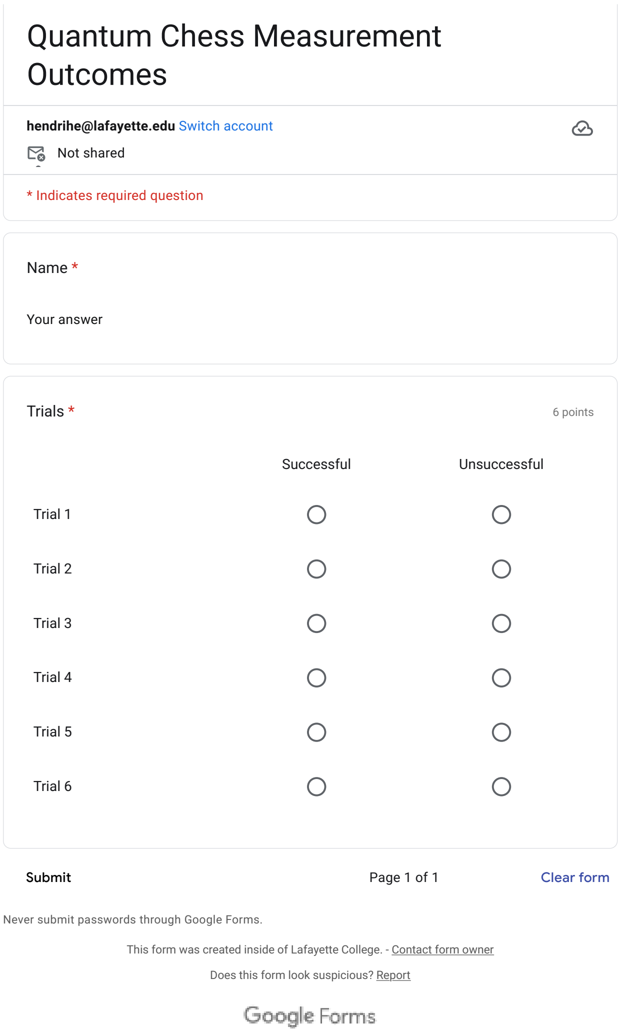

Quantum Chess Measurement Outcomes

hendrihe@lafayette.edu Switch account

Not shared

* Indicates required question

Name *

Your answer

Trials * 6 points

	Successful	Unsuccessful
Trial 1	○	○
Trial 2	○	○
Trial 3	○	○
Trial 4	○	○
Trial 5	○	○
Trial 6	○	○

Submit Page 1 of 1 Clear form

Never submit passwords through Google Forms.

This form was created inside of Lafayette College. - Contact form owner

Does this form look suspicious? Report

Google Forms

Figure 1: Participants entered the results of their measurement activity trials into a Google form so that the results could be compiled and shared with the full group.

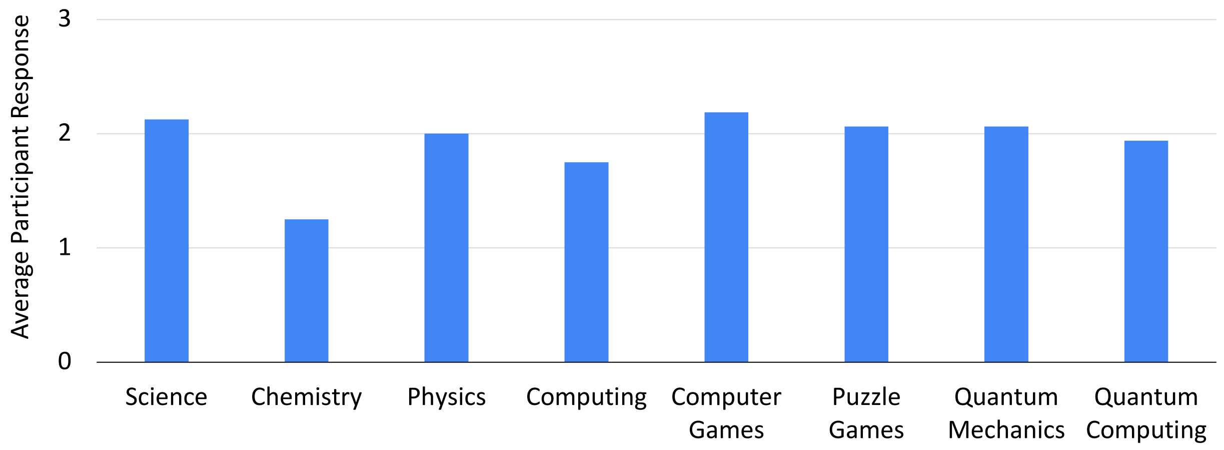

Figure 2: The average participant responses when asked about their interest level in the different subjects related to the workshop.

Although they were interested in these topics, participants reported that they had little to no prior knowledge of either quantum mechanics or quantum computing, as shown in Figure 3.

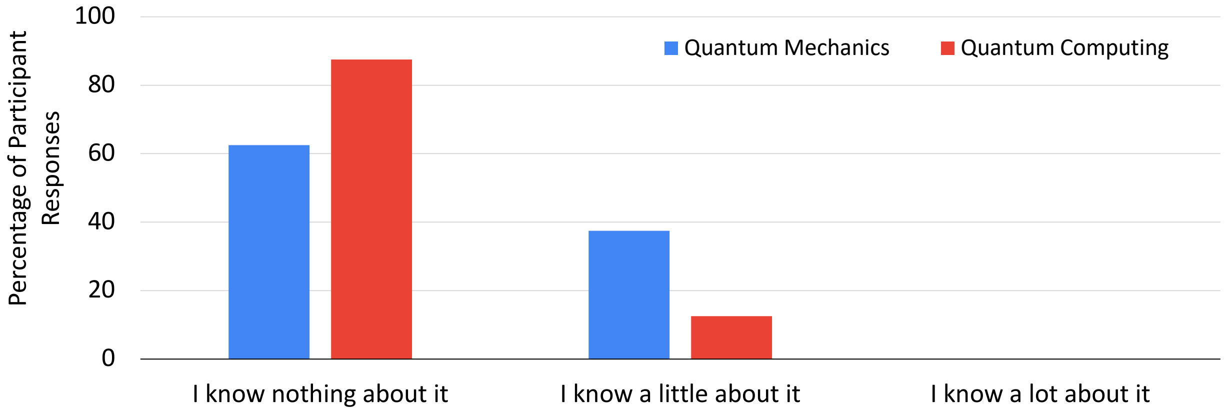

Figure 3: Percentage of participants reporting their degree of prior knowledge of quantum mechanics (blue) and quantum computing (red).

References

(1) Quantum Realm Games. https://quantumrealmgames.com/, [Accessed 03-07-2024].

(2) Cantwell, C. Quantum Chess: Developing a Mathematical Framework and Design Methodology for Creating Quantum Games. 2019; https://arxiv.org/abs/1906.05836.

(3) Nielsen, M. A.; Chuang, I. L. *Quantum Computation and Quantum Information: 10th Anniversary Edition*; Cambridge University Press: Cambridge, 2011.
