# Supplementary material for: Quantum Chess as a Pedagogical Tool for Teaching Quantum Information Science in High Schools
Source: J Chem Educ. 2026 Jun 8;103(7):3971–80. doi: 10.1021/acs.jchemed.5c00836 (PMC13374102; doi:10.1021/acs.jchemed.5c00836)
Supplement: Supplementary file 2 [file ed5c00836_si_002.pdf]

Quantum Chess as a Pedagogical Tool for Teaching Quantum Information Science in High Schools

Padmanabh Kaushik,[†,‡] Nam P. Vu,[†,¶] Crystal Yeung,[†] Swetha Tadisina,[†] Leah Boyle,[†] Vedit Venkatesh,[†] Maya Zilberstein,[†] Nicholas Sorak,[†] Kusum Subedi,[†] Delmar G. A. Cabral,[§] Brandon Allen,[§] Victor S. Batista,[*,§,‖] and Heidi P. Hendrickson[*,†]

†*Department of Chemistry, Lafayette College, Easton, PA 18042, USA*

‡*Department of Biomedical Engineering, Faculty of Engineering and Information Technology, University of Melbourne, Victoria 3010, Australia*

¶*Department of Electrical Engineering and Computer Science, Massachusetts Institute of Technology, Cambridge, MA 02139, USA*

§*Department of Chemistry, Yale University, New Haven, CT 06520, USA*

‖*Yale Quantum Institute, Yale University, New Haven, CT 06511, USA*

E-mail: victor.batista@yale.edu; hendrihe@lafayette.edu

Demonstration of Quantum Computing Concepts via Quantum Chess

1 How to Play Chess?

1.1 How to setup the board?

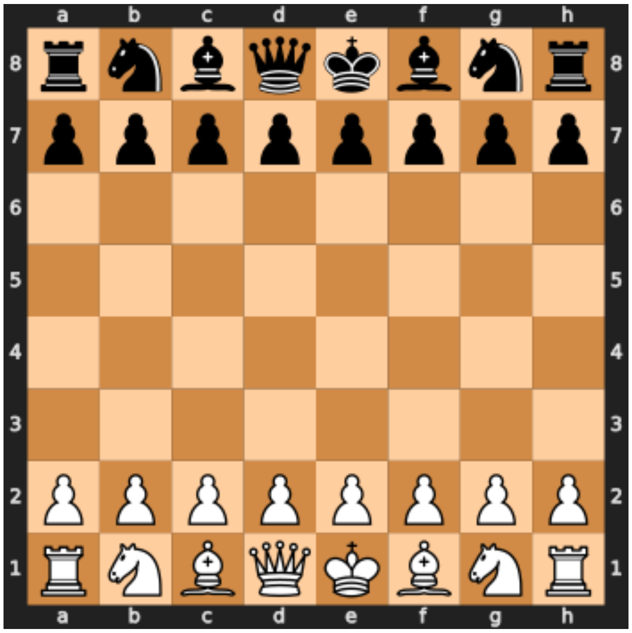

Figure 1: The initial position in a complete chess game.

- 16 pieces for each player
- Players alternate play turns and can only make one move
- Each trying to trap and capture the opposite king (checkmate)
- Often easier by one player have more pieces than the other
- Goal: Achieve a positional/material advantage to perform checkmate

1.2 How to move the pieces?

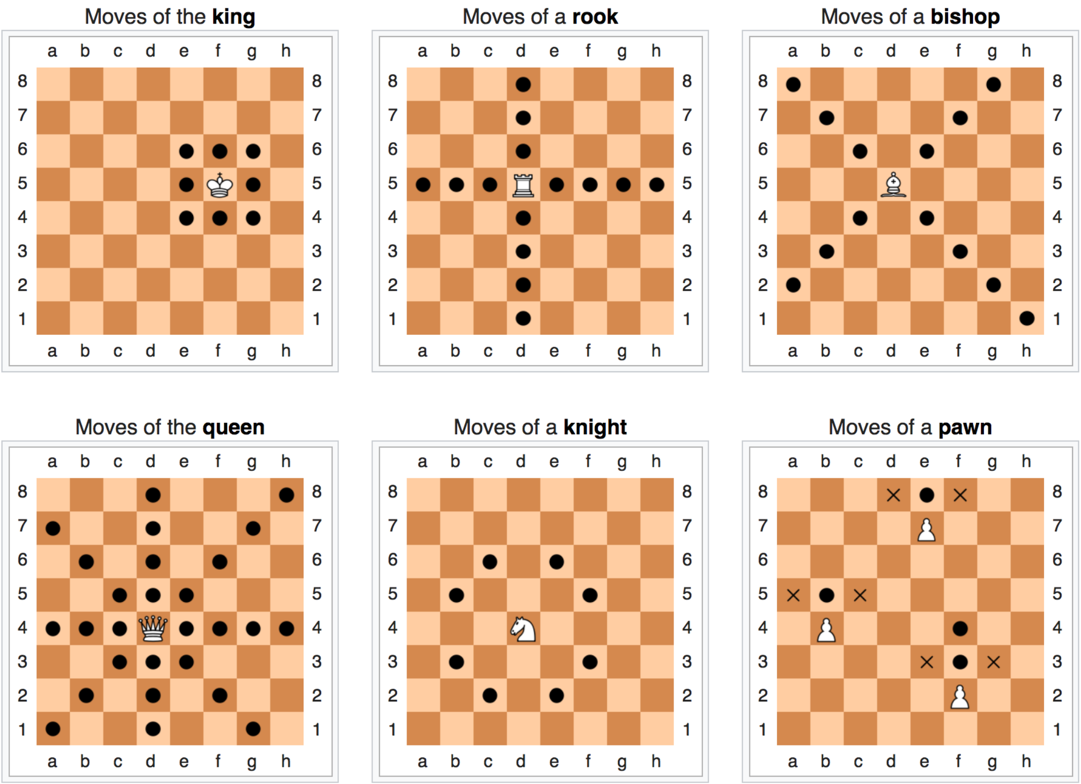

Figure 2: The allowed movements for each piece type in the game of chess. Capturing a piece can be done by moving into a square of an opposing player's piece.

1.3 How to capture?

Capture by occupying the same square as a piece of the opposite player. When and how to capture is an important part of strategy:

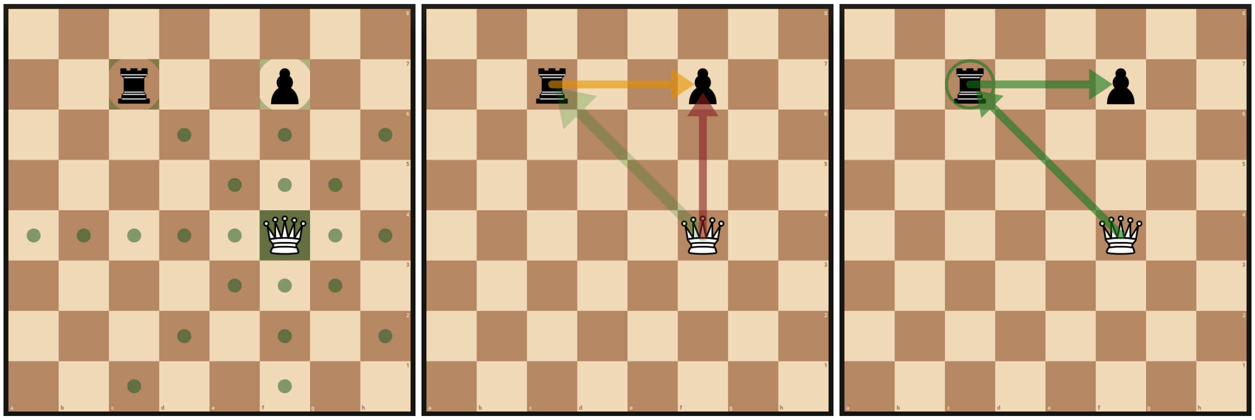

1.4 What is checkmate?

To win, one must threaten to capture the opposite king, while it has no spaces to escape. This is illustrated in figure 3, using a queen and king to deliver the checkmate. Because kings must not be under threat in any gameturn, they also cannot be adjacent to each other.

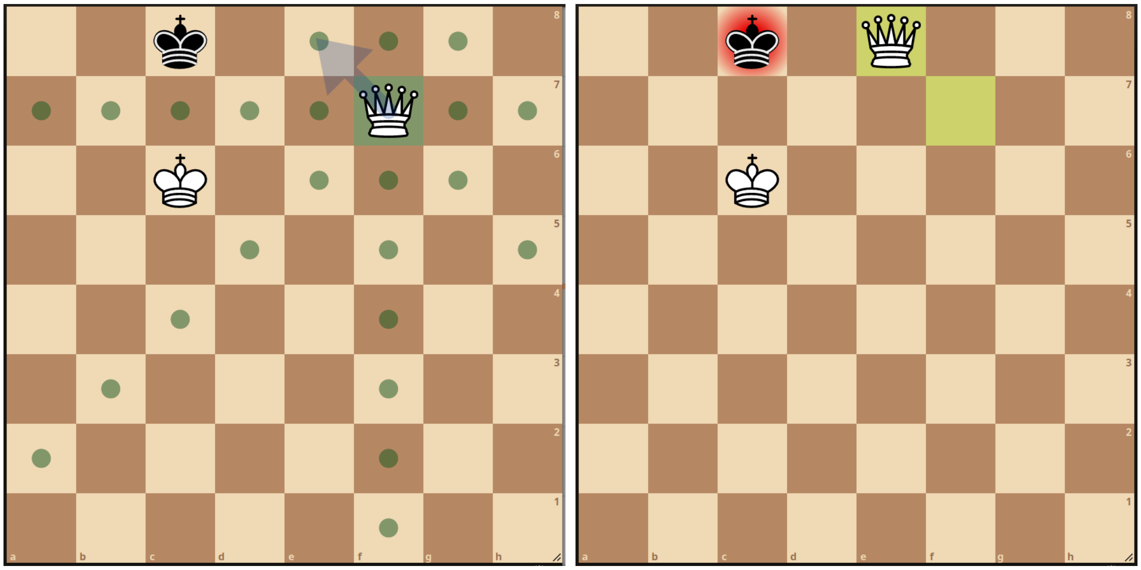

Figure 3: Left: One move before checkmate; the queen's moves are highlighted, with the arrow indicating a move that delivers checkmate. Right: A final checkmate position: the dark king cannot move towards the white king (as kings cannot be adjacent in a game of chess). Furthermore the white queen is threatening capture of the king (hence the red highlight). The king cannot move left or right due to the queen also threatening capturing in those squares.
