# Supplementary material for: Quantum Chess as a Pedagogical Tool for Teaching Quantum Information Science in High Schools
Source: J Chem Educ. 2026 Jun 8;103(7):3971–80. doi: 10.1021/acs.jchemed.5c00836 (PMC13374102; doi:10.1021/acs.jchemed.5c00836)
Supplement: Supplementary file 3 [file ed5c00836_si_003.pdf]

Quantum Chess as a Pedagogical Tool for Teaching Quantum Information Science in High Schools

Padmanabh Kaushik,[†,‡] Nam P. Vu,[†,¶] Crystal Yeung,[†] Swetha Tadisina,[†] Leah Boyle,[†] Vedit Venkatesh,[†] Maya Zilberstein,[†] Nicholas Sorak,[†] Kusum Subedi,[†] Delmar G. A. Cabral,[§] Brandon Allen,[§] Victor S. Batista,[*,§,‖] and Heidi P. Hendrickson[*,†]

†*Department of Chemistry, Lafayette College, Easton, PA 18042, USA*

‡*Department of Biomedical Engineering, Faculty of Engineering and Information Technology, University of Melbourne, Victoria 3010, Australia*

¶*Department of Electrical Engineering and Computer Science, Massachusetts Institute of Technology, Cambridge, MA 02139, USA*

§*Department of Chemistry, Yale University, New Haven, CT 06520, USA*

‖*Yale Quantum Institute, Yale University, New Haven, CT 06511, USA*

E-mail: victor.batista@yale.edu; hendrihe@lafayette.edu

NSF Center for Chemical Innovation

Transformative Quantum Technology for Innovation in Chemistry (CHE-2124511)

Center for Quantum Dynamics on Modular Quantum Devices

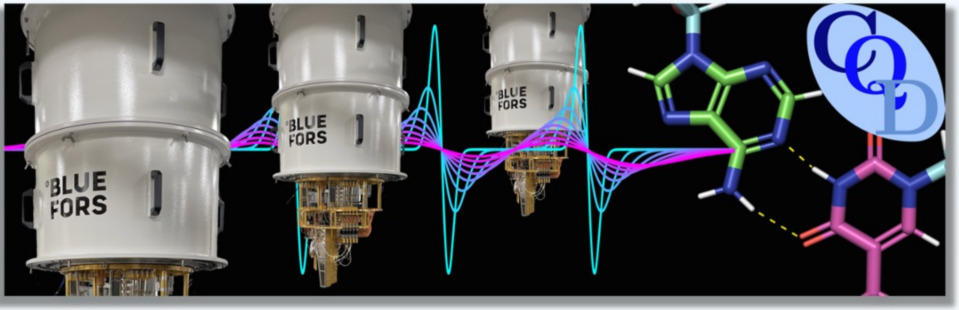

Quantum Games for Quantum Computing

High school name here

April 2024

https://cqdmqd.yale.edu

YouTube Channel

Hendrickson Group

LAFAYETTE COLLEGE

Welcome!

- Who we are:
  - The NSF Center for Chemical Innovation: Center for Quantum Dynamics on Modular Quantum Devices

  - Developing innovative quantum technologies to drive chemistry research forward

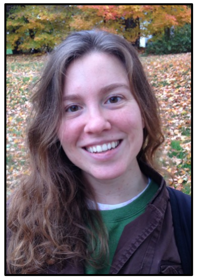

Prof. Heidi Hendrickson

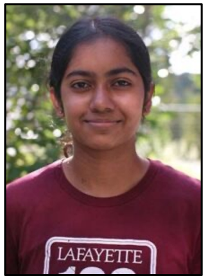

Swetha Tadisina

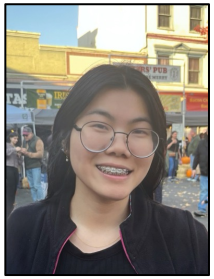

Crystal Yeung

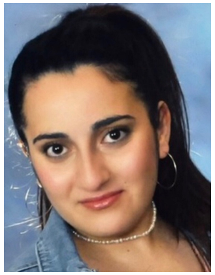

Maya Zilberstein

Workshop Schedule

- Complete Intro Survey (10 min)
- Introduction Quantum Information Science
- Demonstration of Quantum Chess Game and Tutorial
- Exploration
  - Solving Quantum Chess Puzzles
- Explanation
  - The Actual Quantum Physics Behind Quantum Chess
- Exploration
  - Solving More Puzzles and Game Play
- Complete Exit Survey (10 min)
- Closing

Please Complete the Intro Survey

QRCode for Pre-Workshop Survey was included here

Introduction to Quantum Mechanics and Quantum Computing

What is Quantum Computing?

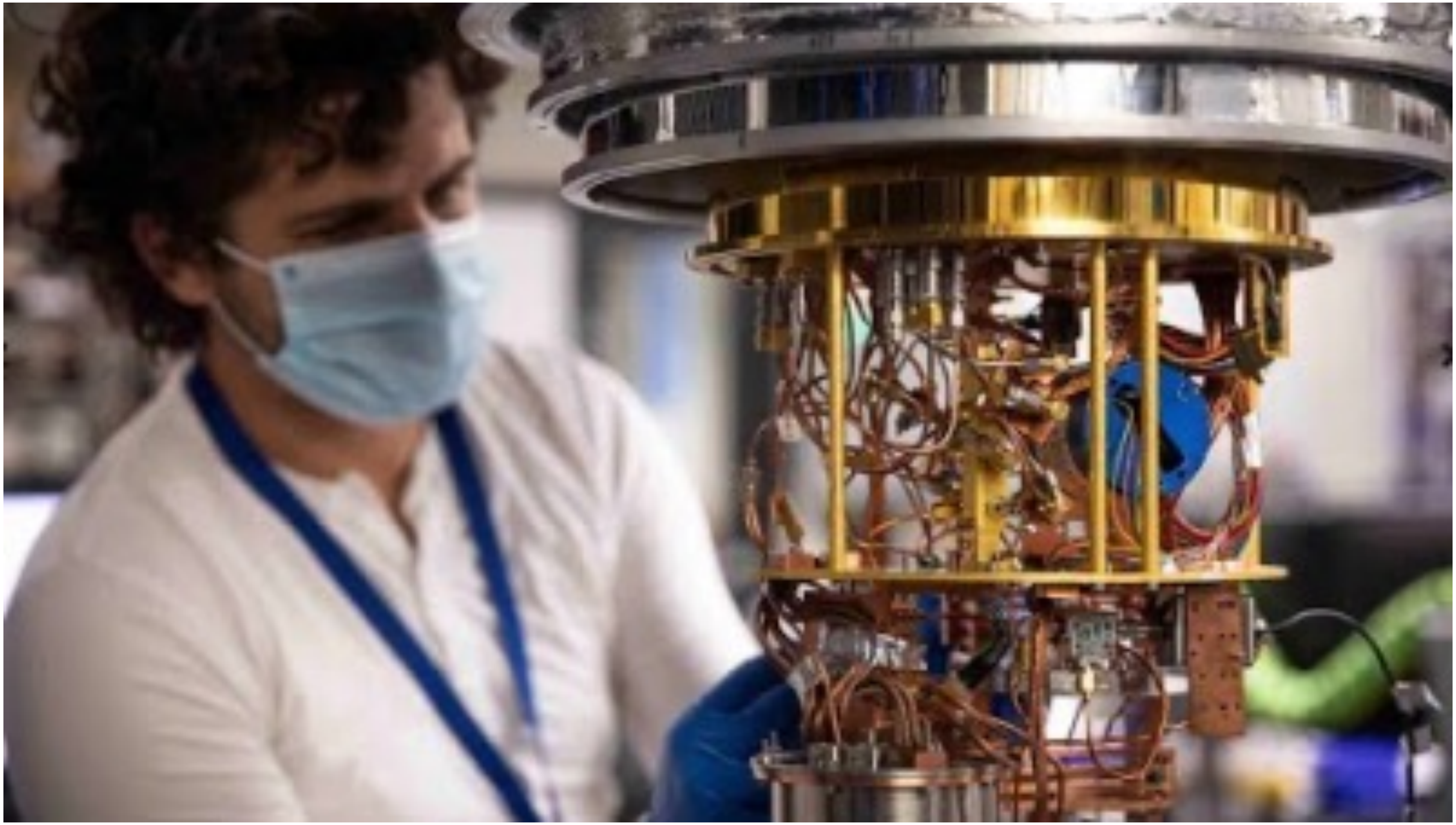

https://www.youtube.com/watch?v=odNaniOvjeI&t=173s

What is a Bit?

- What is a *bit* of information?
  - Information can be coded into a *bit*
  - A bit contains "binary" information:
    - There are two possible values (therefore the Latin prefix "bi")
      - Off or On
      - Heads or Tails
      - 0 or 1

What is a Bit?

- What is a *bit* of information?
  - Information can be coded into a *bit*
  - A bit contains "binary" information:
    - There are two possible values (therefore the Latin prefix "bi")
      - Off or On
      - Heads or Tails
      - 0 or 1
  - Representation
    - Two possible "states"
    - Each state is represented as:

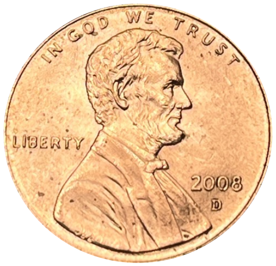

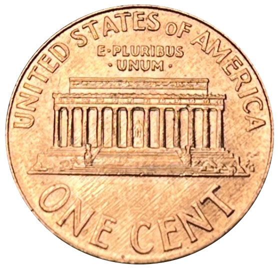

$|0\rangle$ or $|1\rangle$

What is a Bit?

- A more general representation uses both states
  - The coefficients determine the overall state
  - For regular bits, the coefficient can only be zero or one

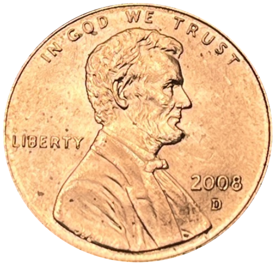

$$|0\rangle$$

What is a Bit?

- A more general representation uses both states
  - The coefficients determine the overall state
  - For regular bits, the coefficient can only be zero or one

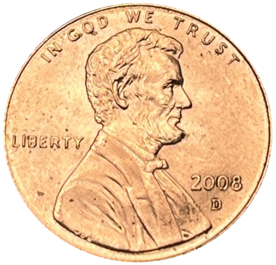

$$|0\rangle$$

$$|0\rangle = 1|0\rangle$$

What is a Bit?

- A more general representation uses both states
  - The coefficients determine the overall state
  - For regular bits, the coefficient can only be zero or one

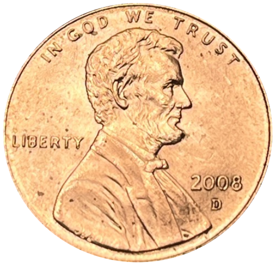

$$|0\rangle$$

$$|0\rangle = 1|0\rangle + 0|1\rangle$$

What is a Bit?

- A more general representation uses both states
  - The coefficients determine the overall state
  - For regular bits, the coefficient can only be zero or one

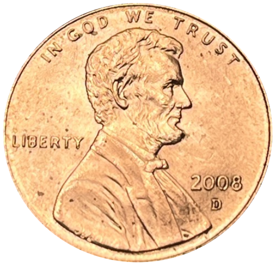

$$|0\rangle$$

$$|0\rangle = 1|0\rangle + 0|1\rangle$$
$$|0\rangle = 1|0\rangle$$

What is a Bit?

- A more general representation uses both states
  - The coefficients determine the overall state
  - For regular bits, the coefficient can only be zero or one

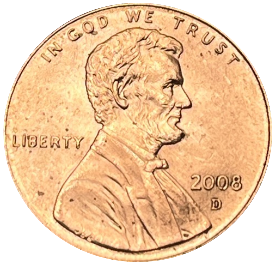

$$|0\rangle$$

$$|0\rangle = 1|0\rangle + 0|1\rangle$$
$$|0\rangle = 1|0\rangle$$
$$|0\rangle = |0\rangle$$

What is a Bit?

- A more general representation uses both states
  - The coefficients determine the overall state
  - For regular bits, the coefficient can only be zero or one

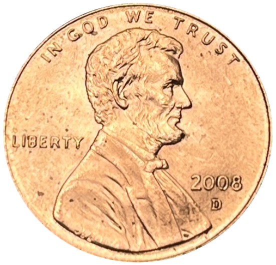

or

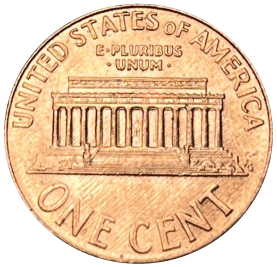

$|0\rangle$ $|1\rangle$

$$|0\rangle = 1|0\rangle + 0|1\rangle$$
$$|0\rangle = 1|0\rangle$$
$$|0\rangle = |0\rangle$$

$$|1\rangle = 1|1\rangle$$

What is a Bit?

- A more general representation uses both states
  - The coefficients determine the overall state
  - For regular bits, the coefficient can only be zero or one

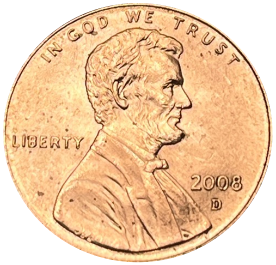

or

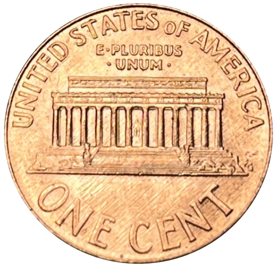

$|0\rangle$ $\qquad$ $|1\rangle$

$$|0\rangle = 1|0\rangle + 0|1\rangle$$
$$|0\rangle = 1|0\rangle$$
$$|0\rangle = |0\rangle$$

$$|1\rangle = 0|0\rangle + 1|1\rangle$$

What is a Bit?

- A more general representation uses both states
  - The coefficients determine the overall state
  - For regular bits, the coefficient can only be zero or one

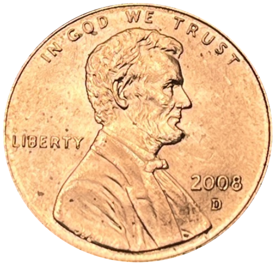

or

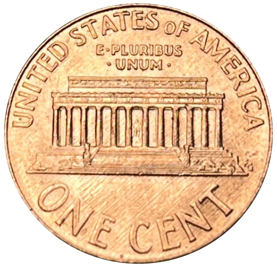

$|0\rangle$ $\qquad$ $|1\rangle$

$$|0\rangle = 1|0\rangle + 0|1\rangle$$
$$|0\rangle = 1|0\rangle$$
$$|0\rangle = |0\rangle$$

$$|1\rangle = 0|0\rangle + 1|1\rangle$$
$$|1\rangle = 1|1\rangle$$

What is a Bit?

- A more general representation uses both states
  - The coefficients determine the overall state
  - For regular bits, the coefficient can only be zero or one

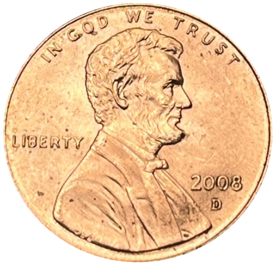

or

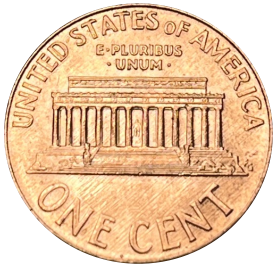

$|0\rangle$

$|1\rangle$

$|0\rangle = 1|0\rangle + 0|1\rangle$
$|0\rangle = 1|0\rangle$
$|0\rangle = |0\rangle$

$|1\rangle = 0|0\rangle + 1|1\rangle$
$|1\rangle = 1|1\rangle$
$|1\rangle = |1\rangle$

What is a Qubit?

- For **quantum bits (qubits)**
  - the coefficients can be **any value** as long as the coefficients squared add up to 1.

We can still have:

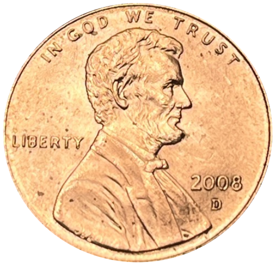

or

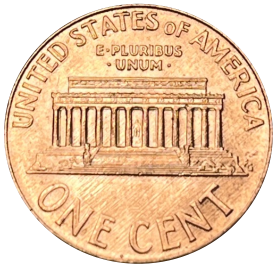

$$|0\rangle = 1|0\rangle + 0|1\rangle$$

$$|1\rangle = 0|0\rangle + 1|1\rangle$$

What is a Qubit?

- For **quantum bits (qubits)**
  - the coefficients can be **any value** as long as the coefficients squared add up to 1.

We can still have:

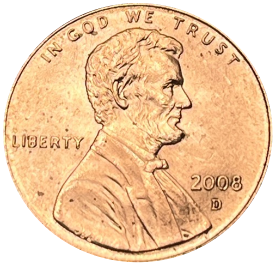

or

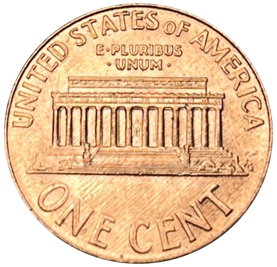

$$|0\rangle = 1|0\rangle + 0|1\rangle$$

$$(1)^2 + (0)^2 = 1 + 0 = 1$$

$$|1\rangle = 0|0\rangle + 1|1\rangle$$

$$(0)^2 + (1)^2 = 0 + 1 = 1$$

What is a Qubit?

- For **quantum bits (qubits)**
  - the coefficients can be **any value** as long as the coefficients squared add up to 1.

But we can also have:

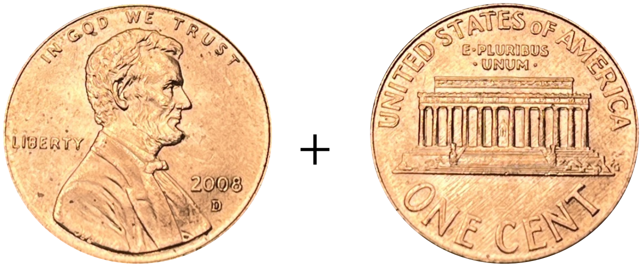

$$|+\rangle = \frac{1}{\sqrt{2}}|0\rangle + \frac{1}{\sqrt{2}}|1\rangle$$

What is a Qubit?

- For **quantum bits (qubits)**
  - the coefficients can be **any value** as long as the coefficients squared add up to 1.

But we can also have:

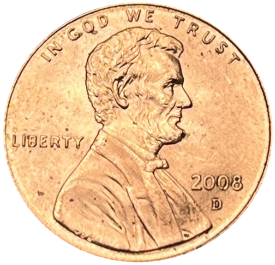

$+$

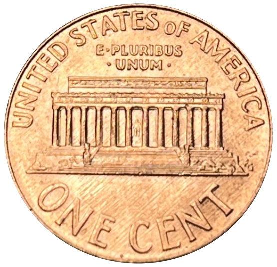

$$|+\rangle = \frac{1}{\sqrt{2}}|0\rangle + \frac{1}{\sqrt{2}}|1\rangle$$

$$\left(\frac{1}{\sqrt{2}}\right)^2 + \left(\frac{1}{\sqrt{2}}\right)^2 =$$

What is a Qubit?

- For **quantum bits (qubits)**
  - the coefficients can be **any value** as long as the coefficients squared add up to 1.

But we can also have:

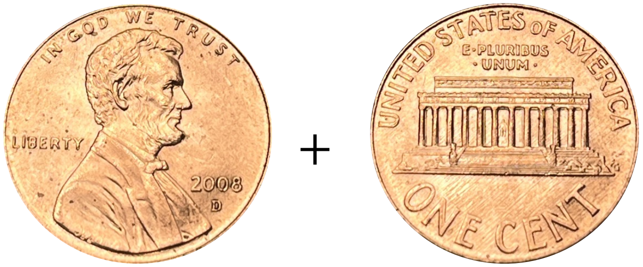

$$|+\rangle = \frac{1}{\sqrt{2}}|0\rangle + \frac{1}{\sqrt{2}}|1\rangle$$

$$\left(\frac{1}{\sqrt{2}}\right)^2 + \left(\frac{1}{\sqrt{2}}\right)^2 = \left(\frac{1^2}{(\sqrt{2})^2}\right) + \left(\frac{1^2}{(\sqrt{2})^2}\right)$$

What is a **Qubit?**

- For **quantum bits (qubits)**
  - the coefficients can be **any value** as long as the coefficients squared add up to 1.

But we can also have:

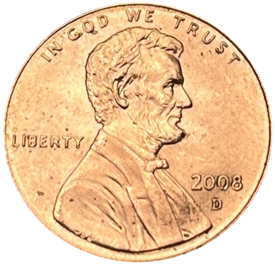

$+$

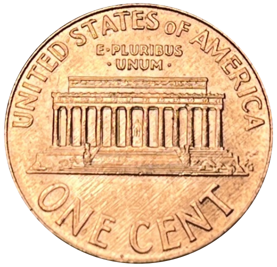

$$|+\rangle = \frac{1}{\sqrt{2}}|0\rangle + \frac{1}{\sqrt{2}}|1\rangle$$

$$\left(\frac{1}{\sqrt{2}}\right)^2 + \left(\frac{1}{\sqrt{2}}\right)^2 = \left(\frac{1^2}{(\sqrt{2})^2}\right) + \left(\frac{1^2}{(\sqrt{2})^2}\right) = \frac{1}{2} + \frac{1}{2}$$

What is a Qubit?

- For **quantum bits (qubits)**
  - the coefficients can be **any value** as long as the coefficients squared add up to 1.

But we can also have:

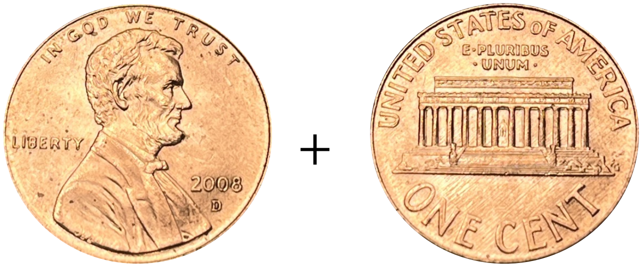

$$|+\rangle = \frac{1}{\sqrt{2}}|0\rangle + \frac{1}{\sqrt{2}}|1\rangle$$

$$\left(\frac{1}{\sqrt{2}}\right)^2 + \left(\frac{1}{\sqrt{2}}\right)^2 = \left(\frac{1^2}{(\sqrt{2})^2}\right) + \left(\frac{1^2}{(\sqrt{2})^2}\right) = \frac{1}{2} + \frac{1}{2} = 1$$

What is a Qubit?

- For **quantum bits (qubits)**
  - the coefficients can be **any value** as long as the coefficients squared add up to 1.

But we can also have:

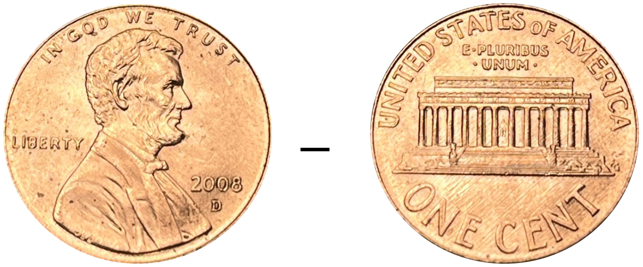

$$|-\rangle = \frac{1}{\sqrt{2}}|0\rangle - \frac{1}{\sqrt{2}}|1\rangle$$

$$\left(\frac{1}{\sqrt{2}}\right)^2 + \left(-\frac{1}{\sqrt{2}}\right)^2 = \left(\frac{1^2}{\left(\sqrt{2}\right)^2}\right) + \left(\frac{(-1)^2}{\left(\sqrt{2}\right)^2}\right) = \frac{1}{2} + \frac{1}{2} = 1$$

What is a Qubit?

- For **quantum bits (qubits)**
  - the coefficients can be **any value** as long as the coefficients squared add up to 1.
  - In general, this type of state is called a ***superposition***

$$|QubitState\rangle = \alpha|State1\rangle + \beta|State2\rangle$$

$$|QubitState\rangle = \alpha|0\rangle + \beta|1\rangle$$

$$(\alpha)^2 + (\beta)^2 = 1$$

What do the coefficients mean?

The coefficient squared in a superposition (i.e., qubit) gives the ***probability*** for finding the qubit in one of the states

- The coefficients squared add up to 100%

$$|+\rangle = \frac{1}{\sqrt{2}}|0\rangle + \frac{1}{\sqrt{2}}|1\rangle$$

What do the coefficients mean?

The coefficient squared in a superposition (i.e., qubit) gives the ***probability*** for finding the qubit in one of the states

- The coefficients squared add up to 100%

$$|+\rangle = \frac{1}{\sqrt{2}}|0\rangle + \frac{1}{\sqrt{2}}|1\rangle$$

$$\left(\frac{1}{\sqrt{2}}\right)^2 + \left(\frac{1}{\sqrt{2}}\right)^2 = \frac{1}{2} + \frac{1}{2} = 0.50 + 0.50 = \boxed{1}$$

$$\left(\frac{1}{\sqrt{2}}\right)^2 + \left(\frac{1}{\sqrt{2}}\right)^2 = \frac{1}{2} + \frac{1}{2} = 50\% + 50\% = \boxed{100\%}$$

What do the coefficients mean?

The coefficient squared in a superposition (i.e., qubit) gives the ***probability*** for finding the qubit in one of the states

- The coefficients squared add up to 100%

$$|+\rangle = \frac{1}{\sqrt{2}}|0\rangle + \frac{1}{\sqrt{2}}|1\rangle$$

$$\left(\frac{1}{\sqrt{2}}\right)^2 + \left(\frac{1}{\sqrt{2}}\right)^2 = \frac{1}{2} + \frac{1}{2} = 0.50 + 0.50 = \boxed{1}$$

$$\left(\frac{1}{\sqrt{2}}\right)^2 + \left(\frac{1}{\sqrt{2}}\right)^2 = \frac{1}{2} + \frac{1}{2} = 50\% + 50\% = \boxed{100\%}$$

If we measure which state the qubit is in,
we have a 50% chance of finding it in state $|0\rangle$
and a 50% chance of finding it in state $|1\rangle$

What do the coefficients mean?

The coefficient squared in a superposition (i.e., qubit) gives the ***probability*** for finding the qubit in one of the states

- The coefficients squared add up to 100%

$$|0\rangle = 1|0\rangle + 0|1\rangle$$

$$(1)^2 + (0)^2 = 1 + 0 = \boxed{1}$$

$$(1)^2 + (0)^2 = 1 + 0 = 100\% + 0\% = \boxed{100\%}$$

If we measure which state the qubit is in,
we have a 100% chance of finding it in state $|0\rangle$
and a 0% chance of finding it in state $|1\rangle$

Quantum Chess Tutorial and Exploration

Hendrickson Group

LAFAYETTE COLLEGE

What is Quantum Chess?

- Like regular chess, but with *superposition* moves!

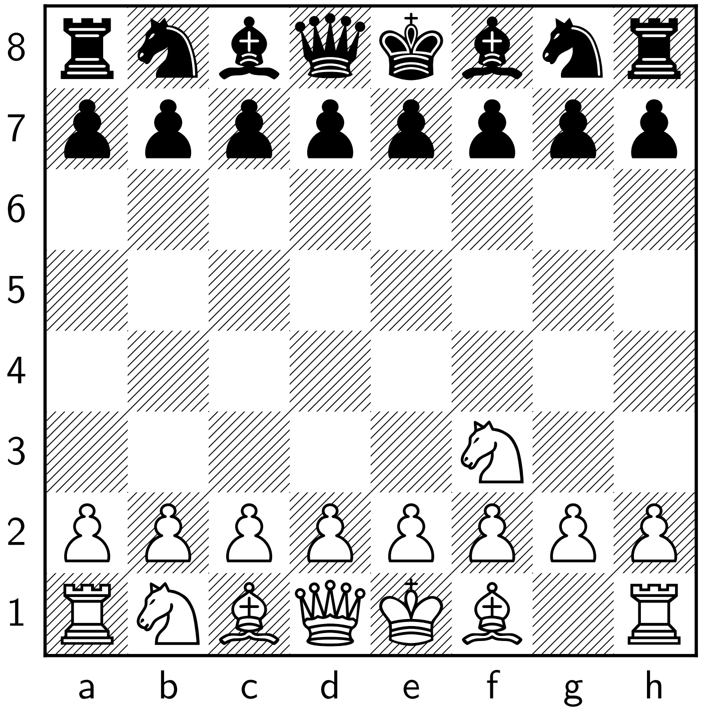

Regular (Classical) Chess

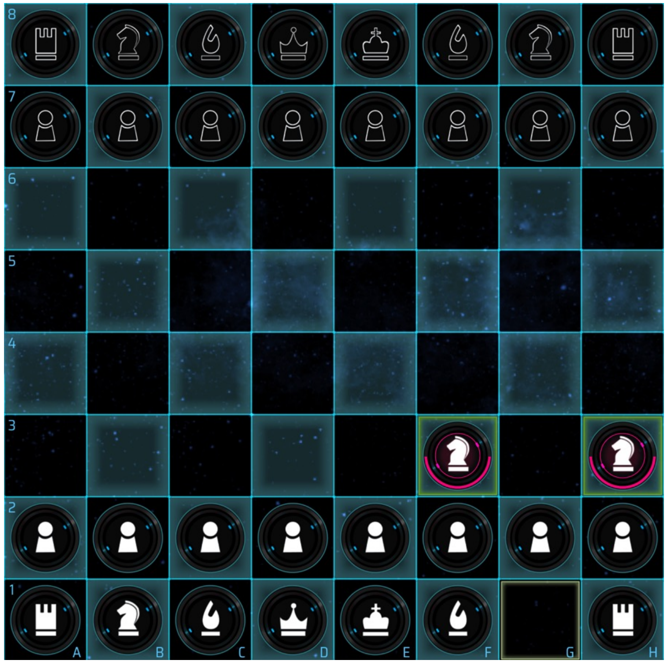

Quantum Chess

C. Cantwell, “Quantum Chess: Developing a Mathematical Framework and Design Methodology for Creating Quantum Games.” arXiv, Jul. 10, 2019. http://arxiv.org/abs/1906.05836

Adapted with permission from Quantum Realm Games. https://quantumrealmgames.com/, Accessed 03-07-2024. Copyright 2025 Quantum Realm Games.

What is Quantum Chess?

- Like regular chess, but with *superposition* moves!

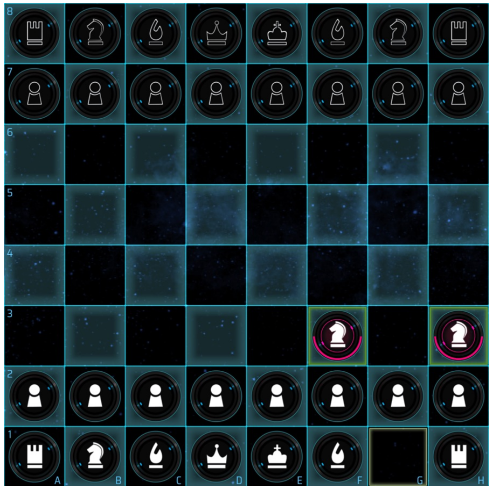

Adapted with permission from Quantum Realm Games. https://quantumrealmgames.com/, Accessed 03-07-2024. Copyright 2025 Quantum Realm Games.

$$= \frac{1}{\sqrt{2}}$$

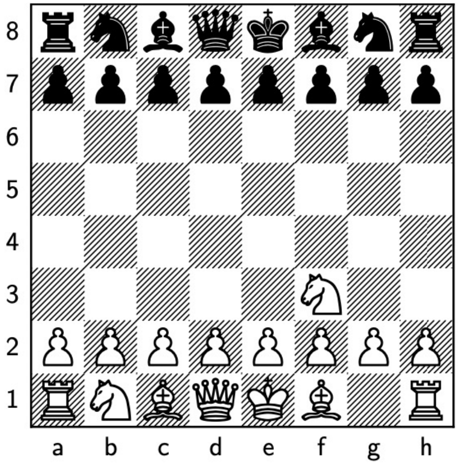

$$+ \frac{1}{\sqrt{2}}$$

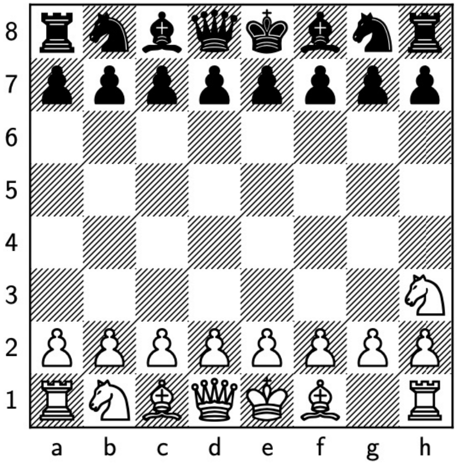

$$\left(\frac{1}{\sqrt{2}}\right)^2 = \frac{1}{2} = 50\%$$

$$\left(\frac{1}{\sqrt{2}}\right)^2 = \frac{1}{2} = 50\%$$

There's a 50% chance the knight is at F3 and a 50% chance the knight is at H3!

Exploration – Let’s play!

- Quantum Chess Game: https://quantumchess.net/play/

Explanation –
How does Quantum Chess relate to Quantum Physics?

Is Superposition Real?

- Is superposition real? Yes!
- Consider how superposition is revealed in **photons** and **electrons**!

- Photons and electrons are quantum particles

- Quantum particles exhibit "wave-particle" duality
  - Particle-like behavior
  - Wave-like behavior
  - Not a particle, not a wave, but something else…

Regular Wave-like behavior

https://phet.colorado.edu/sims/html/wave-interference/latest/wave-interference_en.html

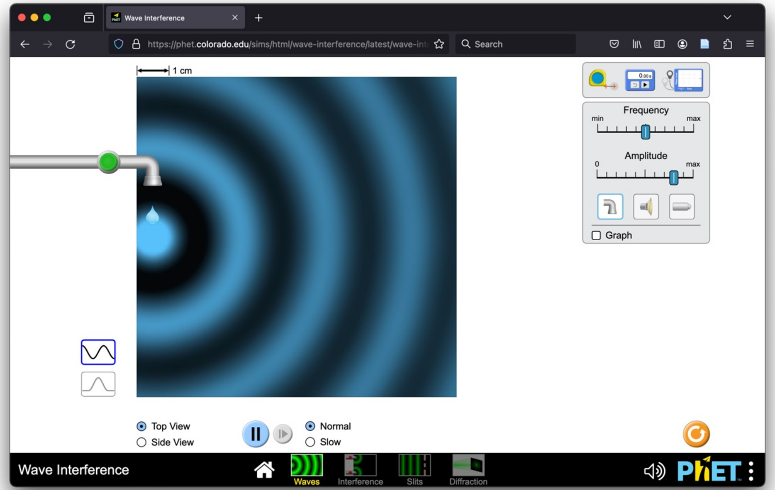

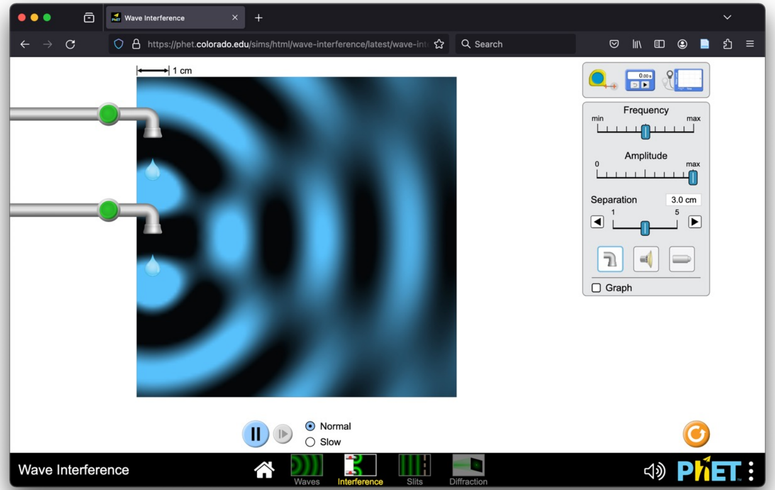

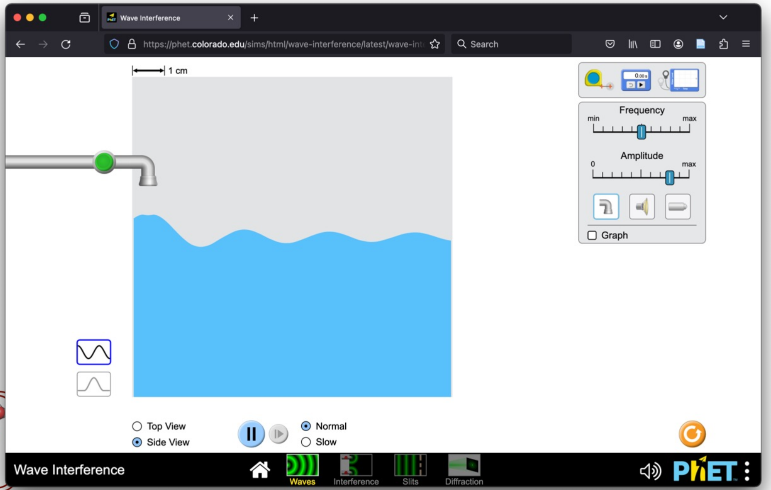

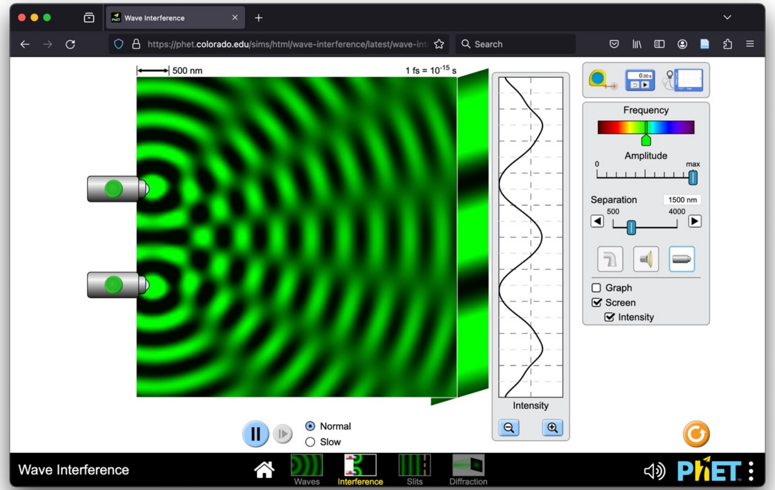

Waves interfere with each other to form standing wave patterns

https://phet.colorado.edu/sims/html/wave-interference/latest/wave-interference_en.html

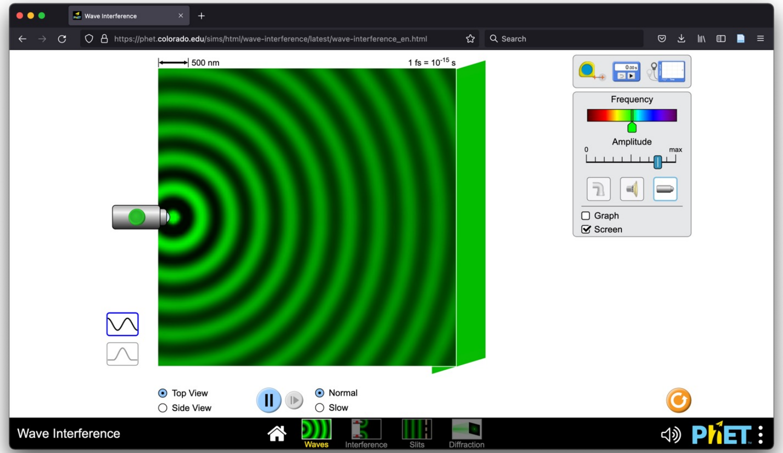

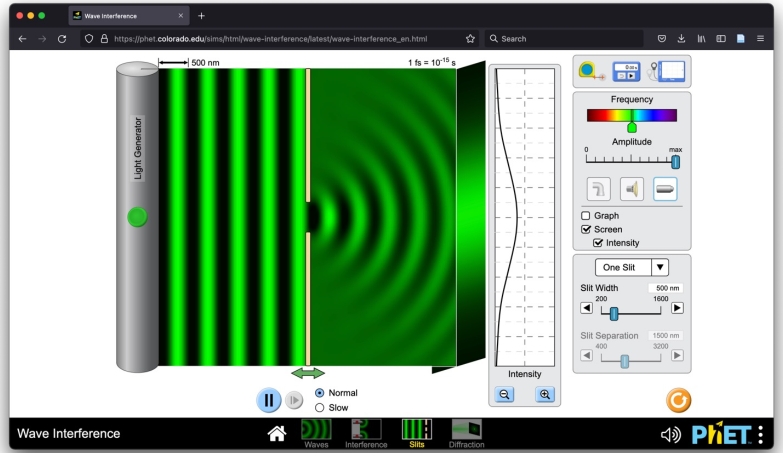

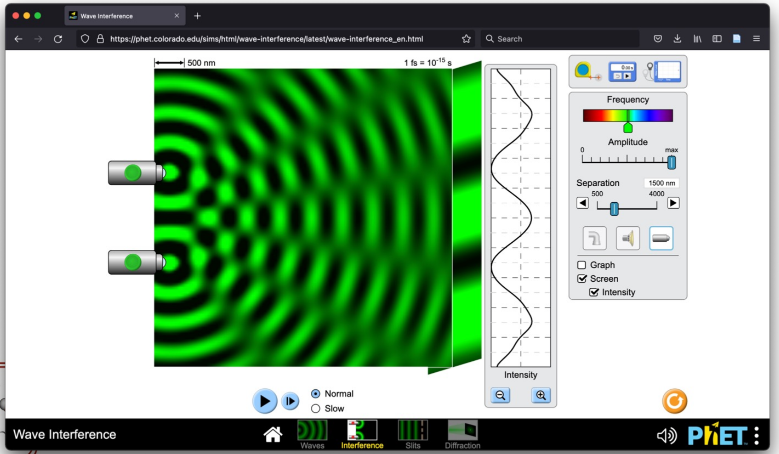

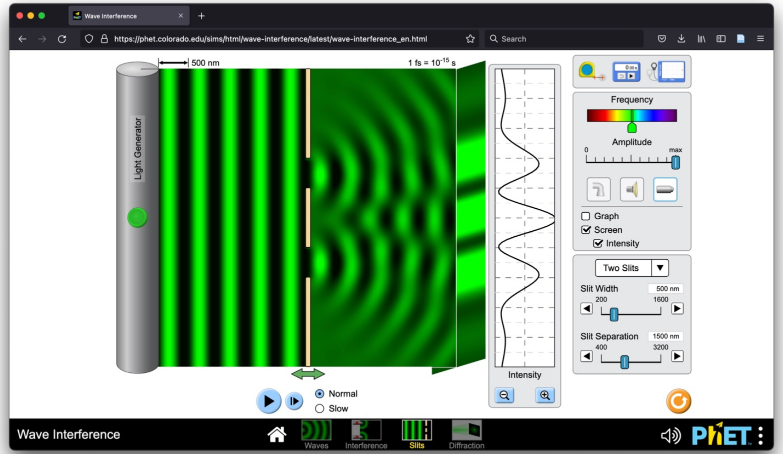

Hendrickson Group

Quantum behavior: Particle-like

https://phet.colorado.edu/sims/cheerpj/quantum-wave-interference/latest/quantum-wave-interference.html?simulation=quantum-wave-interference

- Photons and electrons hit the detection screen one at a time, like particles.

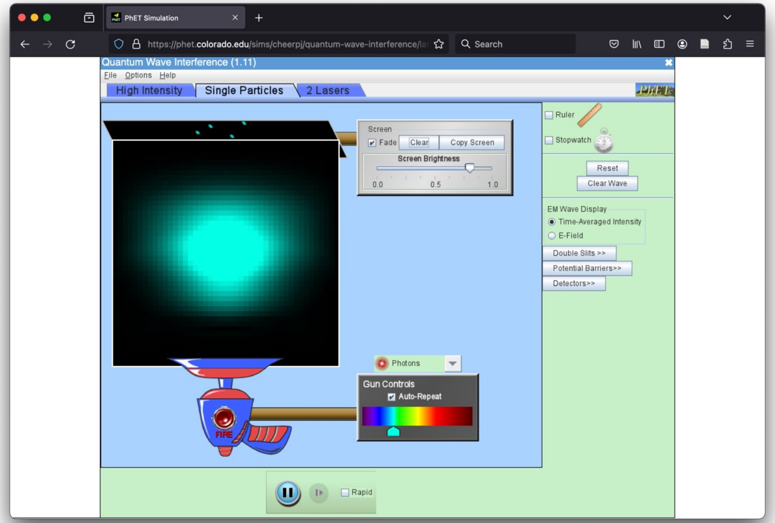

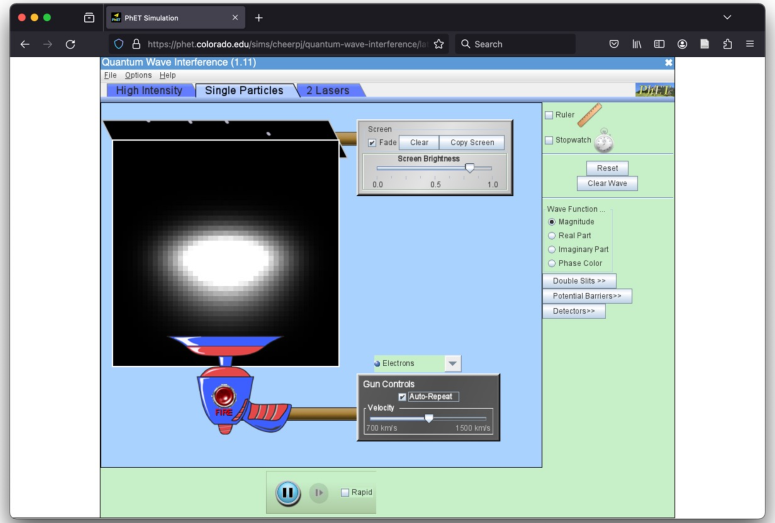

Quantum behavior: Wave-like

https://phet.colorado.edu/sims/cheerpj/quantum-wave-interference/latest/quantum-wave-interference.html?simulation=quantum-wave-interference

- A photon or electron will interfere with itself when traveling through a double-slit apparatus.
- An interference pattern is formed by individual ”particles” hitting the detection screen after interference

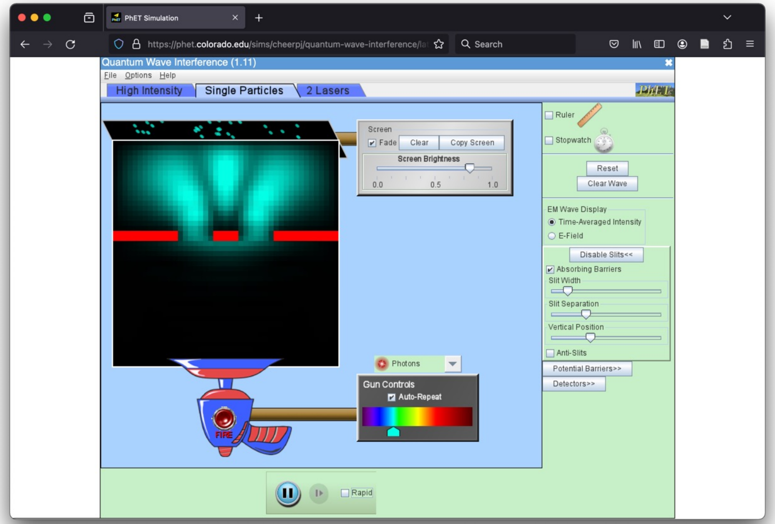

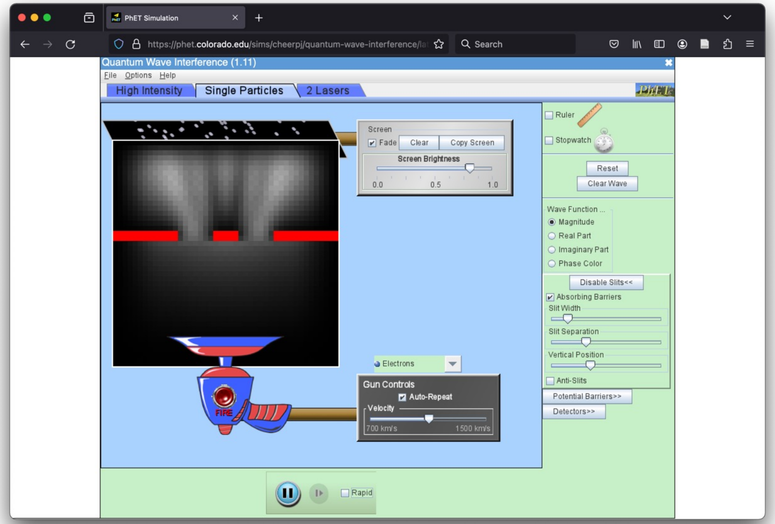

Real Evidence of Superposition

https://phet.colorado.edu/sims/cheerpj/quantum-wave-interference/latest/quantum-wave-interference.html?simulation=quantum-wave-interference

- The electron going through double slits is in a superposition of traveling through slit 1 and slit 2

$$|DoubleSlit\rangle = \frac{1}{\sqrt{2}}|Slit1\rangle + \frac{1}{\sqrt{2}}|Slit2\rangle$$

- There is a 50% chance the electron goes through slit 1 and 50% chance the electron goes through slit 2

Can we measure where the electron went?

https://phet.colorado.edu/sims/cheerpj/quantum-wave-interference/latest/quantum-wave-interference.html?simulation=quantum-wave-interference

- If we measure which slit the electron goes through:
  - The interference pattern is not observed
  - The screen shows particles that went through one slit or the other

Quantum Measurement

https://phet.colorado.edu/sims/cheerpj/quantum-wave-interference/latest/quantum-wave-interference.html?simulation=quantum-wave-interference

- Quantum measurement is probabilistic
  - There is a chance of getting any one of the options within the superposition.

Quantum Measurement

https://phet.colorado.edu/sims/cheerpj/quantum-wave-interference/latest/quantum-wave-interference.html?simulation=quantum-wave-interference

- Quantum measurement is probabilistic
  - There is a chance of getting any one of the options within the superposition.
- Quantum measurement causes *wavefunction collapse*
  - Once you measure, the superposition is destroyed…
  - **Wavefunction collapse**: when the superposition is reduced to one of the options within the superposition

Quantum Measurement

https://phet.colorado.edu/sims/cheerpj/quantum-wave-interference/latest/quantum-wave-interference.html?simulation=quantum-wave-interference

- Quantum measurement is probabilistic
  - There is a chance of getting any one of the options within the superposition.
- Quantum measurement causes *wavefunction collapse*
  - Once you measure, the superposition is destroyed…
  - **Wavefunction collapse**: when the superposition is reduced to one of the options within the superposition

$$|DoubleSlit\rangle = \frac{1}{\sqrt{2}}|Slit1\rangle + \frac{1}{\sqrt{2}}|Slit2\rangle$$

goes to

$$|Slit1\rangle \quad \text{or} \quad |Slit2\rangle$$

Quantum Measurement

https://phet.colorado.edu/sims/cheerpj/quantum-wave-interference/latest/quantum-wave-interference.html?simulation=quantum-wave-interference

No measurement
Interference pattern observed

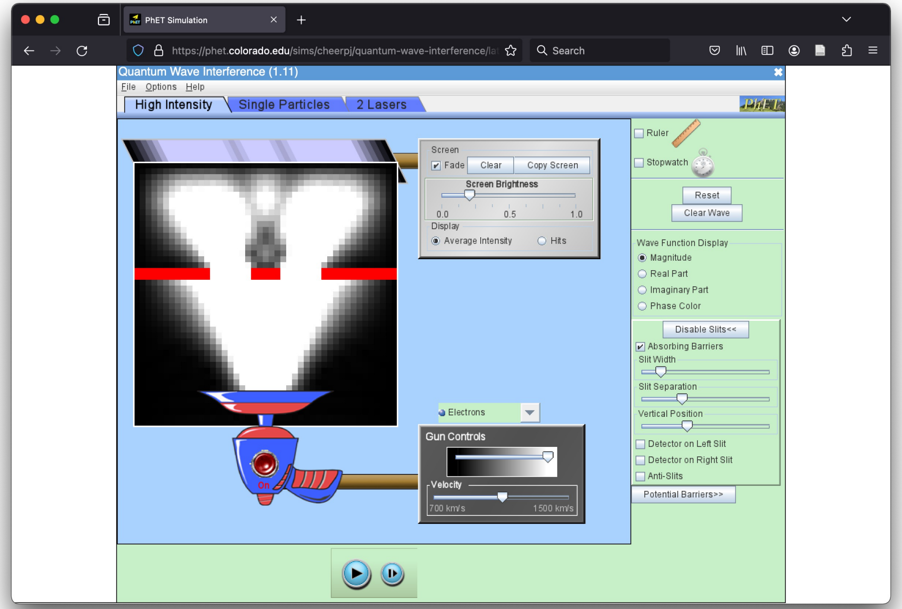

Measurement
No interference pattern observed

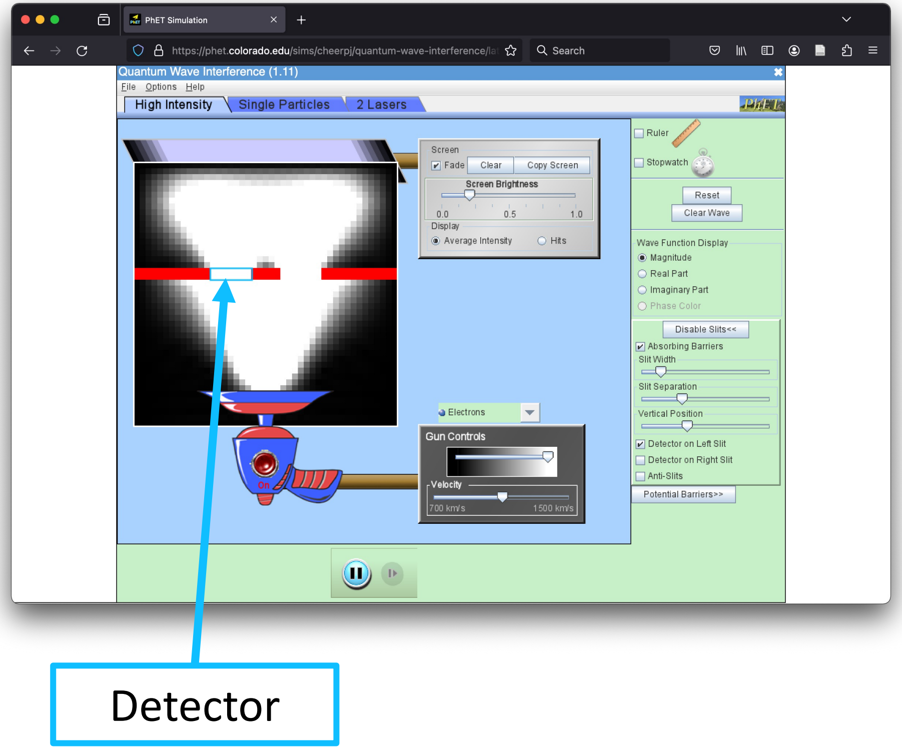

Quantum Measurement Activity

Quantum Measurement Activity

- Start with Puzzle 2 – Quantum Fork

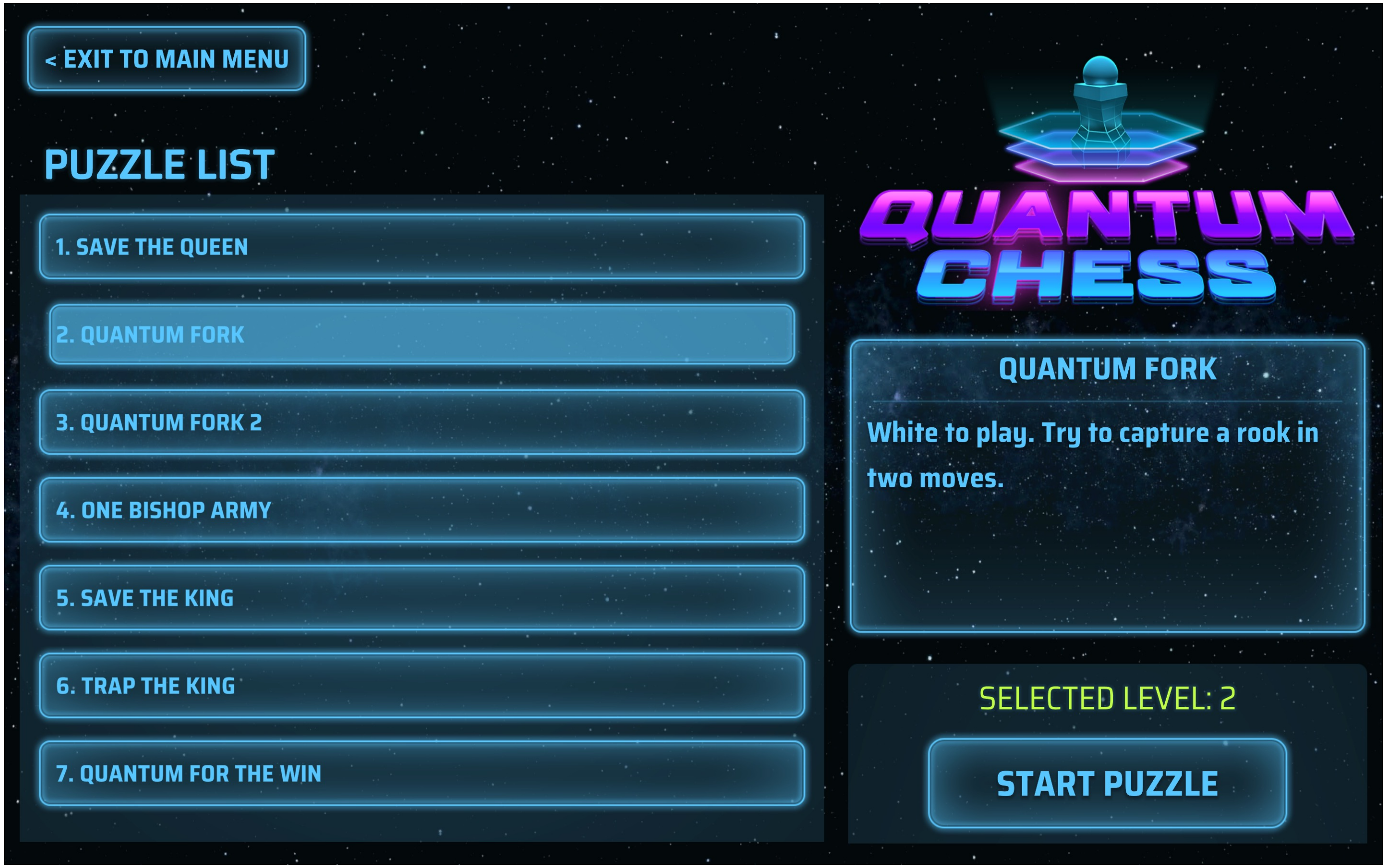

Hendrickson Group

Adapted with permission from Quantum Realm Games. https://quantumrealmgames.com/, Accessed 03-07-2024. Copyright 2025 Quantum Realm Games.

Step 1: Quantum Fork

- Create a superposition state for the knight on B5 and E2

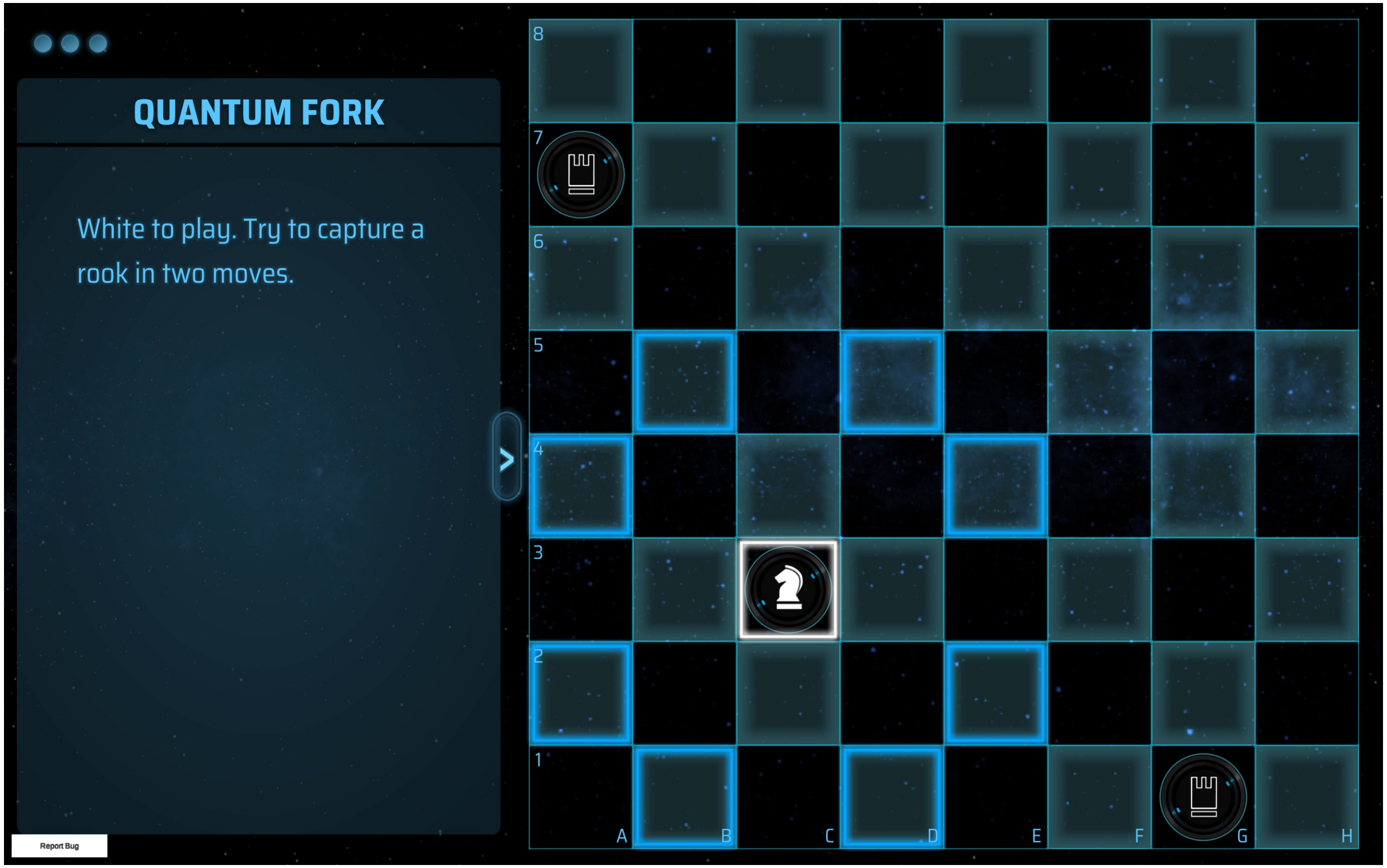

Hendrickson Group

Adapted with permission from Quantum Realm Games. https://quantumrealmgames.com/, Accessed 03-07-2024. Copyright 2025 Quantum Realm Games.

Step 1: Quantum Fork

- Create a superposition state for the knight on B5 and E2

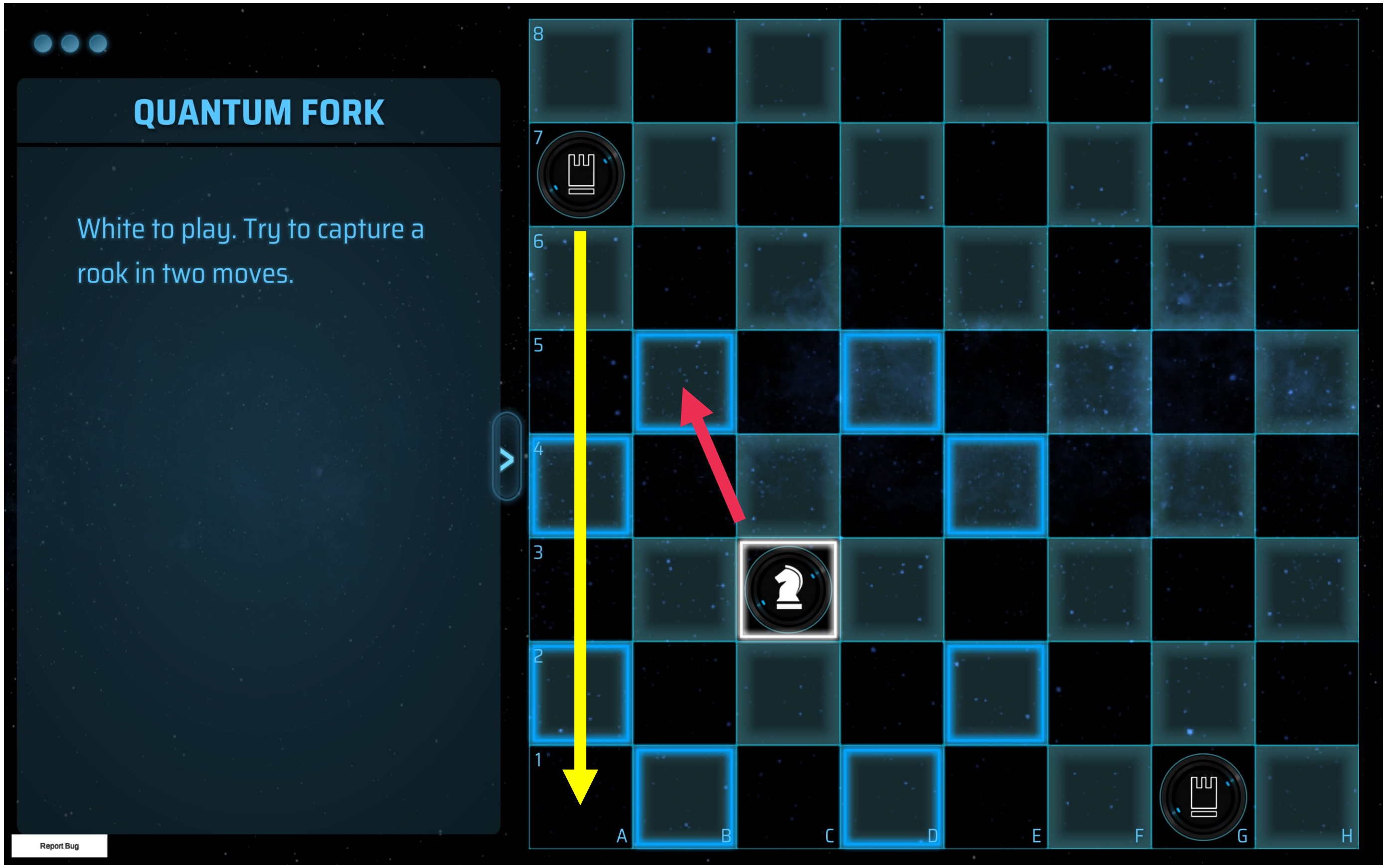

Hendrickson Group

Adapted with permission from Quantum Realm Games. https://quantumrealmgames.com/, Accessed 03-07-2024. Copyright 2025 Quantum Realm Games.

Step 1: Quantum Fork

- Create a superposition state for the knight on B5 and E2

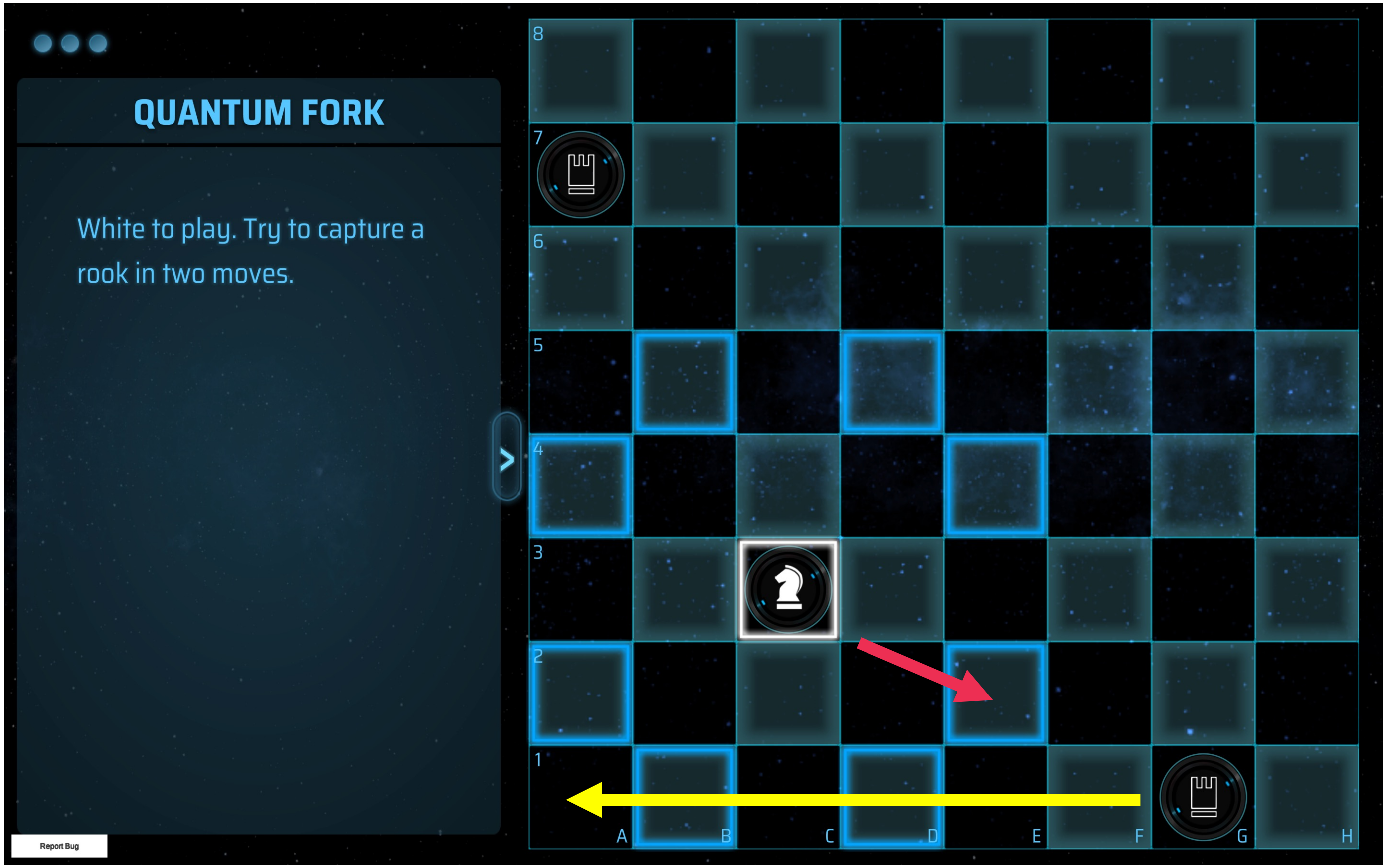

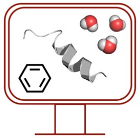

Adapted with permission from Quantum Realm Games. https://quantumrealmgames.com/, Accessed 03-07-2024. Copyright 2025 Quantum Realm Games.

Step 2: Quantum Fork

- Create a superposition state for the knight on B5 and E2

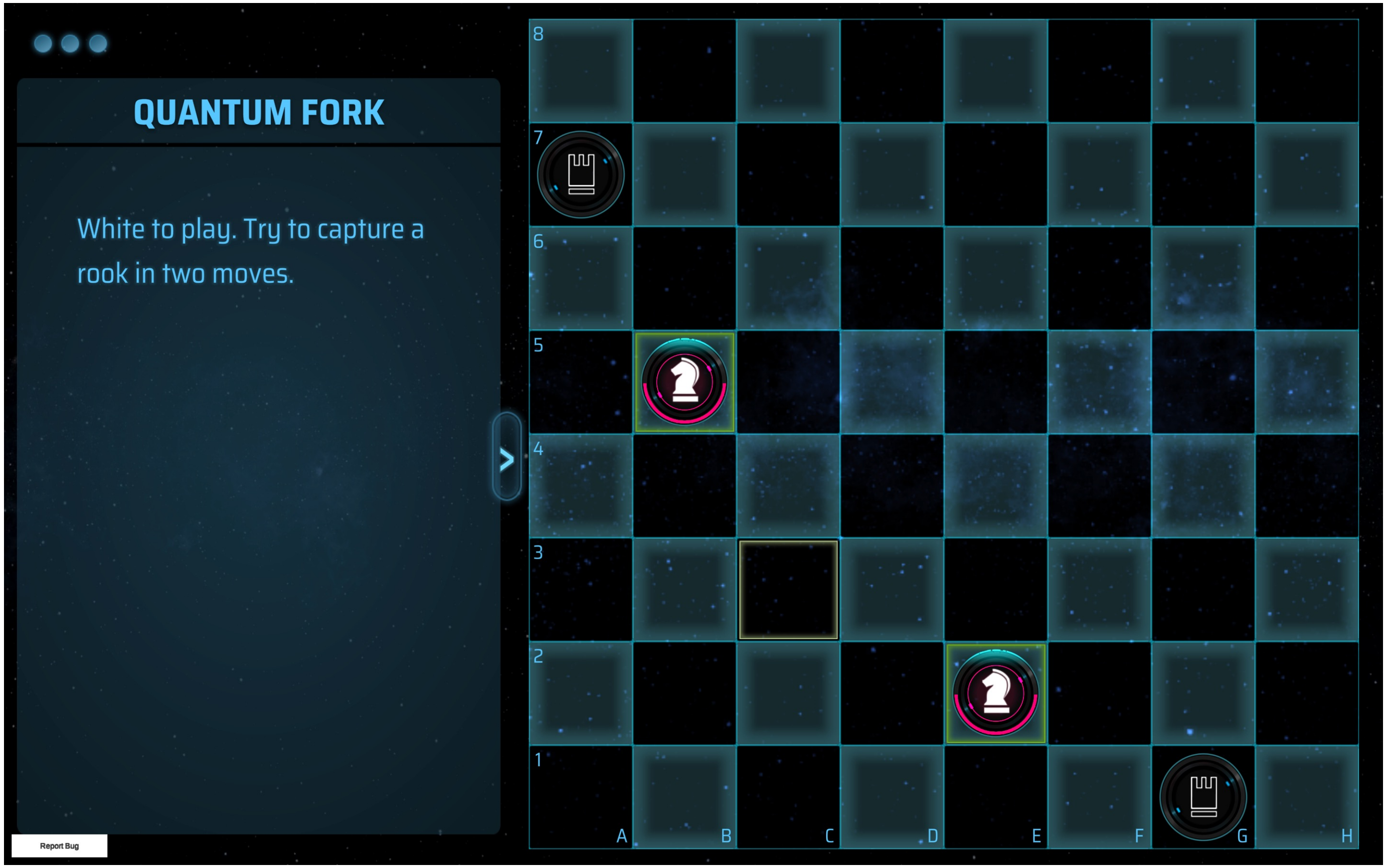

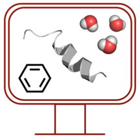
Hendrickson Group

Adapted with permission from Quantum Realm Games. https://quantumrealmgames.com/, Accessed 03-07-2024. Copyright 2025 Quantum Realm Games.

Step 3: Quantum Fork

- Try to capture the rook at G1 using the knight at E2

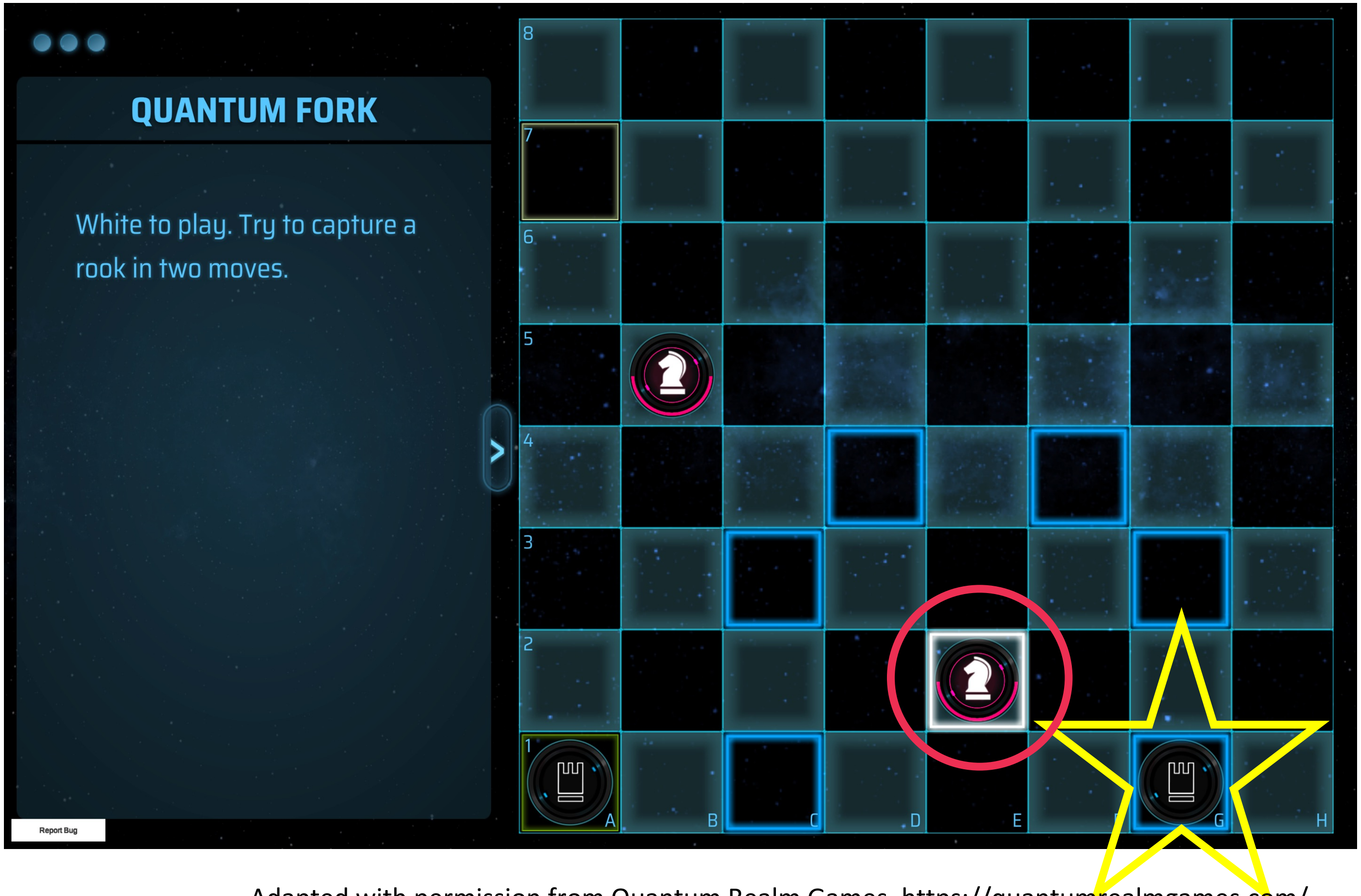

Adapted with permission from Quantum Realm Games. https://quantumrealmgames.com/, Accessed 03-07-2024. Copyright 2025 Quantum Realm Games.

Making Quantum Measurements

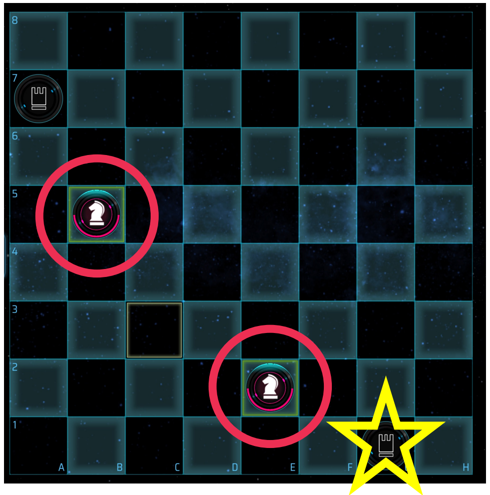

$=\frac{1}{\sqrt{2}}$

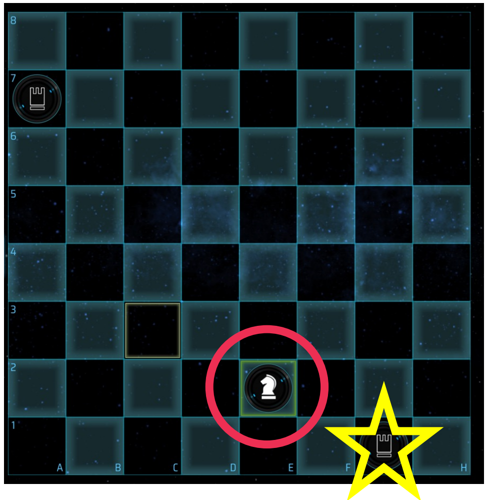

$+\frac{1}{\sqrt{2}}$

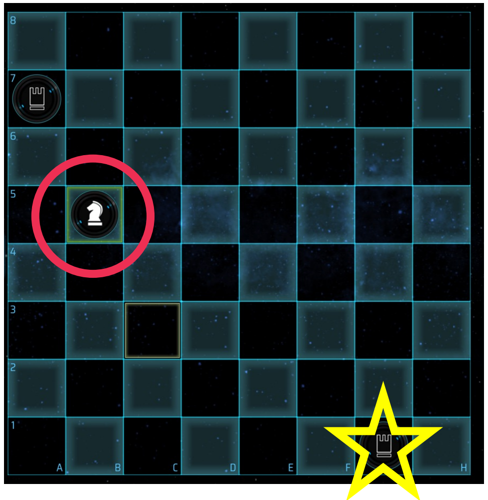

Determining where the knight is located on the board is like ***measuring*** the superposition state

There's a 50% chance the knight is at E2

The quantum move will succeed in capturing the rook!

There's a 50% chance the knight is at B5

The quantum move will fail to capture the rook…

Hendrickson Group

Adapted with permission from Quantum Realm Games. https://quantumrealmgames.com/, Accessed 03-07-2024. Copyright 2025 Quantum Realm Games.

Quantum Measurement Outcomes

- Two outcomes are possible for the correct quantum move!

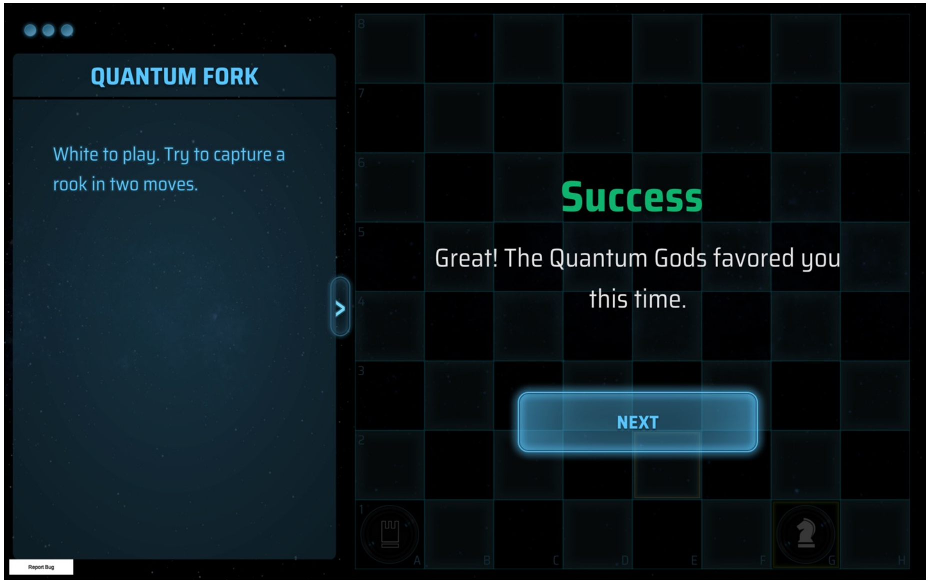

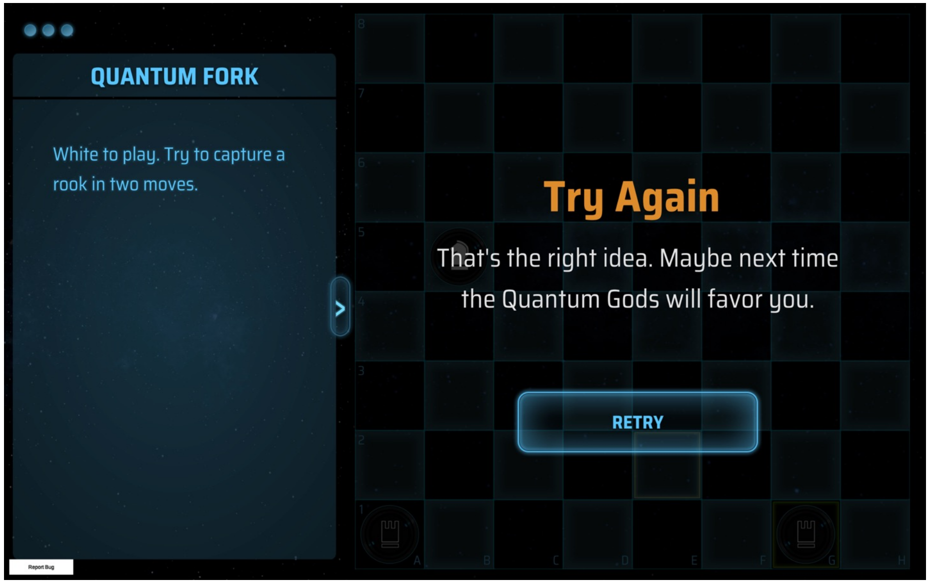

There's a 50% chance the knight is at E2 and a 50% chance the knight is at B5!

In quantum measurement of a superposition state, the outcome will always be ***only one*** of the individual states in the superposition

Hendrickson Group

Adapted with permission from Quantum Realm Games. https://quantumrealmgames.com/, Accessed 03-07-2024. Copyright 2025 Quantum Realm Games.

Let’s see how it works!

- Run 6 trials of Quantum Fork on your own
- Record the results of each trial using the Google Form
  - Only count outcomes that are correct quantum move

SUCCESSFUL OUTCOME

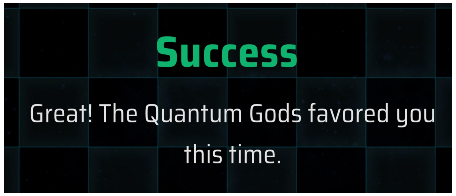

UNSUCCESSFUL OUTCOME

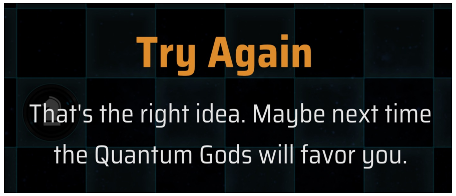

  - Do not count outcomes that use the wrong move

NOT A VALID OUTCOME

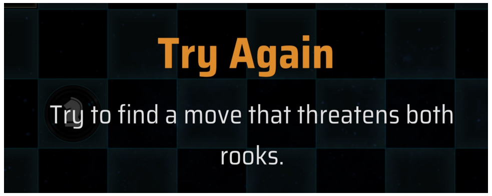

NOT A VALID OUTCOME

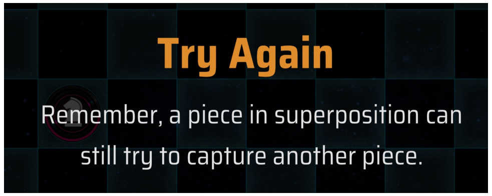

Hendrickson Group

Adapted with permission from Quantum Realm Games. https://quantumrealmgames.com/, Accessed 03-07-2024. Copyright 2025 Quantum Realm Games.

Example Results

- It's most likely that 50% of the trials are successful – as expected!
- It's still possible to get other outcomes…

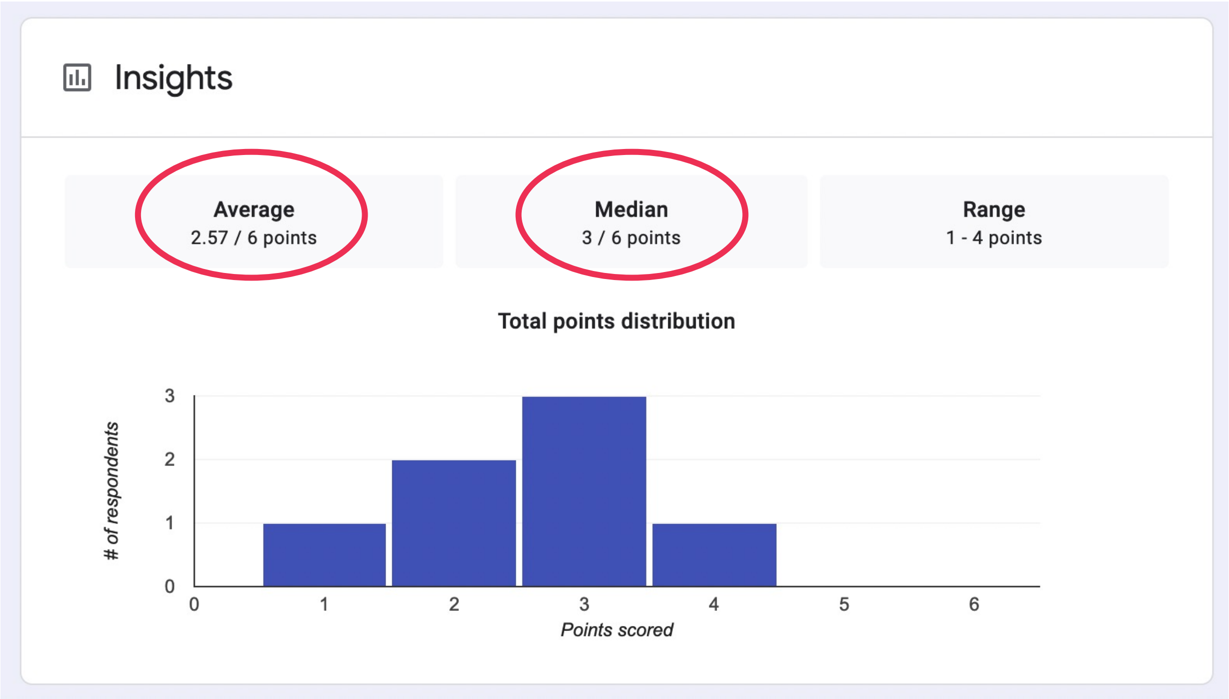

Workshop Summary

- A Qubit is a quantum superposition of two states

$$|QubitState\rangle = \alpha|0\rangle + \beta|1\rangle$$

$$|+\rangle = \frac{1}{\sqrt{2}}|0\rangle + \frac{1}{\sqrt{2}}|1\rangle$$

- The coefficients squared for each state in a superposition must add up to 100%

$$(\alpha)^2 + (\beta)^2 = 100\%$$

$$\left(\frac{1}{\sqrt{2}}\right)^2 + \left(\frac{1}{\sqrt{2}}\right)^2 = \frac{1}{2} + \frac{1}{2} = 50\% + 50\% = 100\%$$

- The outcome of a quantum measurement is always ***just one*** of the states in the superposition
  - The outcome of a measurement of the $|+\rangle$ Qubit state is either $|0\rangle$ or $|1\rangle$
  - The coefficient squared for each state is the probability of measuring that state

  - For the the $|+\rangle$ Qubit state:
    - 50% chance of measuring state $|0\rangle$
    - 50% chance of measuring state $|1\rangle$

The Power of Quantum Computing

- Multiple bits can be coded to represent more complicated information
  - Four bits coded for numbers 0 to 15

Number	Bits
0	$\|0000\rangle$
1	$\|0001\rangle$
2	$\|0010\rangle$
3	$\|0011\rangle$
4	$\|0100\rangle$
5	$\|0101\rangle$
6	$\|0110\rangle$
7	$\|0111\rangle$

Number	Bits
8	$\|1000\rangle$
9	$\|1001\rangle$
10	$\|1010\rangle$
11	$\|1011\rangle$
12	$\|1100\rangle$
13	$\|1101\rangle$
14	$\|1110\rangle$
15	$\|1111\rangle$

- Consider how much more is possible using Qubit superpositions!

What can Quantum Computers Do?

Materials discovery and drug development

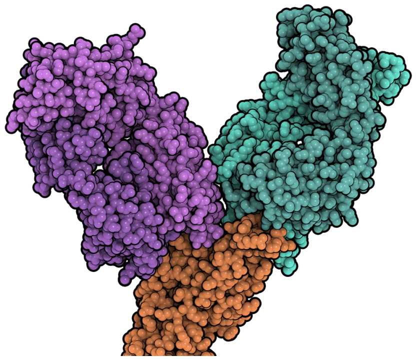

By Fvasconcellos (talk · contribs) - From PDB entry 7L7E. More information:Dong J, Zost SJ, Greaney AJ, Starr TN, Dingens AS, Chen EC (2021). "Genetic and structural basis for SARS-CoV-2 variant neutralization by a two-antibody cocktail.". Nat Microbiol 6 (10): 1233-1244. DOI:10.1038/s41564-021-00972-2. PMID 34548634. PMC: 8543371., Public Domain, https://commons.wikimedia.org/w/index.php?curid=112515054

Cybersecurity

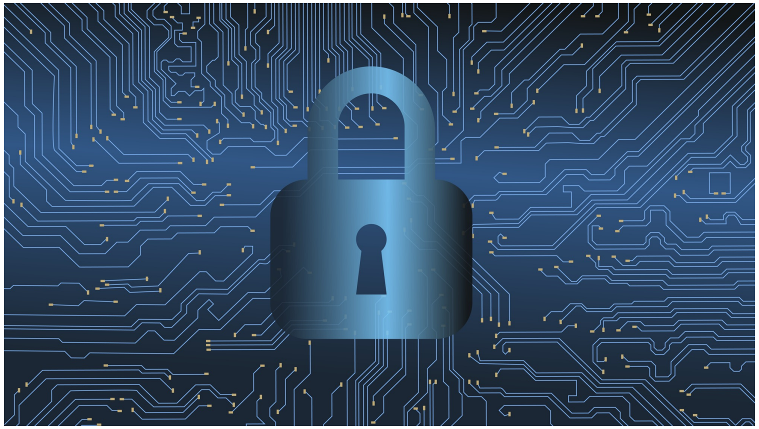

By jaydeep_ - https://pixabay.com/en/hacking-cybercrime-cybersecurity-3112539/ archive copy at the Wayback Machine, CC0, https://commons.wikimedia.org/w/index.php?curid=69573226

Artificial Intelligence

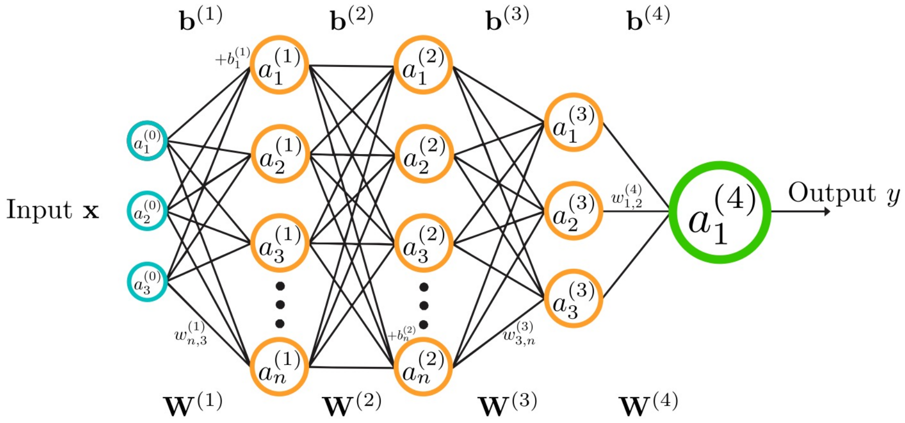

By QuantuMechaniX8 - Own work, CC0, https://commons.wikimedia.org/w/index.php?curid=166026387

Finance and Big Data

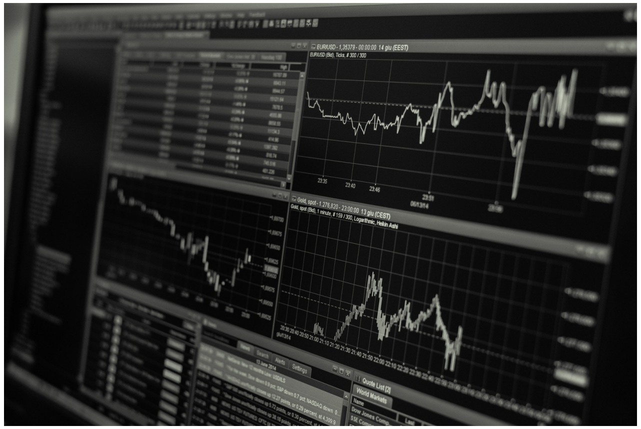

By Unknown author - https://pxhere.com/en/photo/1170475, CC0, https://commons.wikimedia.org/w/index.php?curid=162739807

Hendrickson Group

Please Complete the Exit Survey

QRCode for Post-Workshop Survey was included here

NSF Center for Chemical Innovation

Transformative Quantum Technology for Innovation in Chemistry (CHE-2124511)

NSF

Center for Quantum Dynamics on Modular Quantum Devices

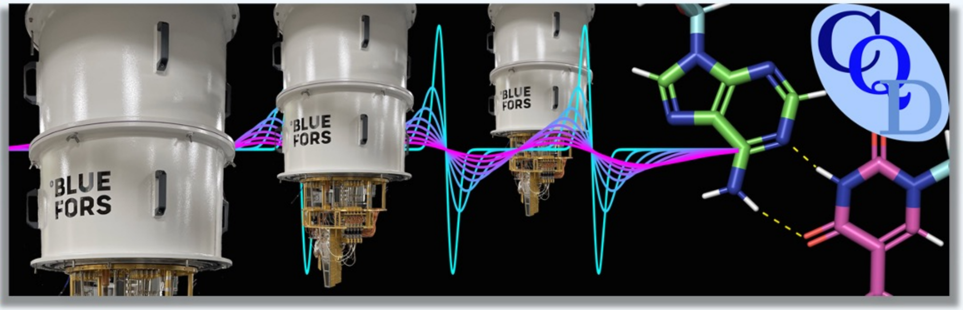

Thank you for participating!

https://cqdmqd.yale.edu

YouTube Channel

Hendrickson Group

LAFAYETTE COLLEGE
