# Supplementary material for: Quantum Chess as a Pedagogical Tool for Teaching Quantum Information Science in High Schools
Source: J Chem Educ. 2026 Jun 8;103(7):3971–80. doi: 10.1021/acs.jchemed.5c00836 (PMC13374102; doi:10.1021/acs.jchemed.5c00836)
Supplement: Supplementary file 4 [file ed5c00836_si_004.pdf]

Quantum Chess as a Pedagogical Tool for Teaching Quantum Information Science in High Schools

Padmanabh Kaushik,[†,‡] Nam P. Vu,[†,¶] Crystal Yeung,[†] Swetha Tadisina,[†] Leah Boyle,[†] Vedit Venkatesh,[†] Maya Zilberstein,[†] Nicholas Sorak,[†] Kusum Subedi,[†] Delmar G. A. Cabral,[§] Brandon Allen,[§] Victor S. Batista,[*,§,‖] and Heidi P. Hendrickson[*,†]

†*Department of Chemistry, Lafayette College, Easton, PA 18042, USA*

‡*Department of Biomedical Engineering, Faculty of Engineering and Information Technology, University of Melbourne, Victoria 3010, Australia*

¶*Department of Electrical Engineering and Computer Science, Massachusetts Institute of Technology, Cambridge, MA 02139, USA*

§*Department of Chemistry, Yale University, New Haven, CT 06520, USA*

‖*Yale Quantum Institute, Yale University, New Haven, CT 06511, USA*

E-mail: victor.batista@yale.edu; hendrihe@lafayette.edu

1 Instructor Guide for Quantum Games for Quantum Computing Workshop

Here we provide presentation notes for instructors who may want to incorporate the workshop into their classes.

Slide 2: Hello everyone and welcome to our workshop on Quantum Games for Quantum Computing! We are a group of researchers collaborating with the NSF Center for Quantum Dynamics on Modular Quantum Devices, and we are working to develop new quantum computing technology that will enable us to solve difficult computational problems in the field of chemistry. We will introduce ourselves ... (introductions)

Slide 3: During our workshop today, we will first ask you to complete an intro survey introduce quantum computing, and you will learn about quantum superpositions and qubits. Then we will move into using the quantum chess game to understand how superpositions work Then we will talk a bit more about how the quantum chess game is related to actual quantum physics And then we will do a quantum measurement activity during which you will learn about probabilistic measurement outcomes And finally, you'll have more time to play Quantum Chess Then we will ask you to complete an exit survey So let's get started!

Slide 5: First we will give a little introduction on quantum computing.

Slide 6: First we will share this video made by our collaborators at Yale, which gives a general overview on quantum computing and why it is exciting!

Slide 7: Ok, so let's go over some of that information in the video, and let's get started talking about what a qubit is... To do that, we need to talk about regular bits. So, what is a bit? Well, it is a bit (or piece) of information. A bit contains binary information, where the "bi" is the Latin prefix for two, so there are two possible values a bit can have. As you saw in the video, that could be like On or off. Or it could be like heads or tails of a coin, or it could just be the zero or one states.

Slide 8: We can represent the two possible states using this special notation here. The

brackets shown here is called a ket, and you may see this notation elsewhere, but basically, this is just a way to represent the two states.

Slide 9: We could also potentially make our representation a bit more general by taking into account both states when writing a single bit state. To do that, we can write the overall state as a weighted sum of the two possible states. But in order for state zero to actually be state zero, we need to make sure that the state zero is multiplied by 1, and that state 1 is multiplied by zero.

Slide 13: If we do that, then when we add together the values in our new notation, we get that state zero equals state zero

Slide 14: And we can also do this for state 1.

Slide 17: Ok, so it seems like we just overcomplicated things, but let's see how this notation helps us understand qubits!

Slide 18: For a regular bit, the coefficients could only be zero or one. In a Qubit however, the coefficients can be any value, so long as the sum of the squares of the coefficients adds up to 1. So like the video said, this means that there are infinitely many qubit states because the coefficients can be any number. For example, we can still have the regular bit states, those are stlll valid.

Slide 19: As you can see the square of the coefficients for the regular bit state add up to 1.

Slide 20: But we can also have states where both coefficients are non-zero! As long as the square of the coefficients adds up to 1, it's all good. You might be wondering why the coefficient looks weird, like 1 over sqrt of 2.

Slide 21 The reason is that if we square the coefficients, we get $\frac{1}{2} + \frac{1}{2}$, and those add up to 1.

Slide 25: And we can also have coefficients with a negative sign, for example.

Slide 26: In general, we can have any value for the coefficients, as shown here. Alpha can be any value, and beta could be any value, so long as the square of the coefficients sum to

1. This type of state that combines multiple states in this way is called a superposition.

Slide 27: So what do these coefficients mean? It turns out, that the square of each coefficient stands for a probability of finding the qubit in the state that corresponds to the coefficient.

Slide 28: So the $\frac{1}{2}$ plus $\frac{1}{2}$ equals 1 really means 50% plus 50% equals 100%

Slide 29: What this means is that if we measure which state the qubit is in, we have a 50% chance of finding it in state 0 and a 50% chance of finding it in state 1

Slide 30: And this is the case for whatever the coefficients are, so it works for the regular 0 or 1 state as well.

Slide 31: Ok, so let's see these superpositions in action! Maya will now lead you through a tutorial on Quantum Chess! (Lead students through quantum chess tutorial)

Slide 32: Now that you have a little idea about quantum superposition, let's see how it is demonstrated in Quantum Chess! In quantum chess, we can use superposition moves! In regular chess, the knight can move up two squares and over one square. So the first move in the game could be to move from G1 to F3, or it could also be possible to move to H3.

Slide 33: In quantum chess, we can create a 50/50 superposition of both of these choices! So in this superposition state, there is a 50% chance the knight is at F3 and a 50% chance the knight is at H3!

Slide 35: First we will give a little introduction on quantum physics.

Slide 36: So the way superpositions work is a little strange, but it is indeed an actual quantum phenomenon that is important for quantum particles like electrons and photons. Electrons are subatomic particles with a negative charge that are involved in chemistry and chemical reactions , and photons are quantum particles of light. We say "quantum particles" because although these entities display particle-like behavior, they also display wave-like behavior! We call that wave-particle duality, but in reality, quantum particles are not really particles, and not really waves, they are something else that we have a hard time understanding intuitively.

Slide 37: Ok, so to understand superposition in quantum particles, we have to consider their wave-like behavior. We can use this demo to understand regular wave behavior, and then use that to see how quantum wave behavior is different. Go ahead and either scan this QR Code or navigate to this website so that you can follow along with the demonstration! (Using the demonstration, walk the students through the aspects of the demonstration. Show them the side view of the wave so that they can better understand the 2D surface representation. Talk about how ripples in the surface of water interfere with each other and cause patterns on the surface of the water while showing the two drops. Then show that this works for other types of waves including light waves. Show that we can see the pattern of interference caused by the superposition by putting up a screen, and we see regions where the light hits the screen and regions where it doesn't. These patterns are expected when there's a superposition wave.)

Slide 38: So now that you can see how superpositions work for waves, let's take a look at a couple of ways we can create these types of superpositions. We can see that if we have a single source of light, we don't get a superposition. So that's all well and good. If we don't have a point source, but we have a plane wave instead, we can still get something that looks more like a point source when the wave passes through a small slit in a barrier. After passing through a slit, the wave acts similar as if it came from a single point source. It's a little different because it's more intense in the middle of the screen, and has less intensity toward the edges of the screen, but it doesn't give us that interference pattern that we saw arise from two point sources. If we add another slit though, so that the barrier has what is called a "double slit", then it's similar to having two point sources when the wave goes through those slits. As a result, we get an interference pattern because the wave after passing through, acts as if it's a superposition wave from two point sources. Now, here we've shown this for light waves, but if you explore the demonstration, you will see that this is the case for water and sound waves as well!

Slide 39: Here is where things get cool when we are dealing with quantum particles

though. So as we said, they have wave-particle duality. They are not really waves, not really particles, because they behave like both. Feel free to navigate to this other quantum demonstration using the website address or the QR Code. We can see the particle like behavior of photons and electrons, because as shown in this demo, when a low-intensity source is used that can shoot out a single photon or electron at a time, we can detect that photon or electron on a screen, similar to the last demo, then we can see that the photon or electron hits the screen one at a time, just like we would expect a particle to do.

Slide 40: However, it's weird because when we shoot that photon or electron at a double slit, it turns out that the SINGLE electron or photon must be interfering with itself, because although a single photon or electron will hit the screen one at a time, after many many photons or electrons hit the screen, the resulting pattern on the screen is an interference pattern. What that means is that while the particle will only land in one place, until it lands, it's not clear exactly where that particle is. It's sort of in a wave-like distribution that looks like an interference pattern of having gone through both slits, just like a wave would do.

Slide 41: The double slit experiment shown in these demos is an actual phenomena that has been observed by scientists! That wavelike interference pattern is actually observed when particles pass through the double slit apparatus. The way that scientists have figured out best explains this is that electrons going through the double slit are in a superposition of traveling through both slits. The form of the superposition looks just like what we saw when we were talking about superpositions in quantum chess! It means that there's a 50% chance the electron goes through slit 1 and a 50% chance the electron goes through slit 2.

Slide 42: Now here's the weird thing! If we measure which slit the electron goes through, then the interference pattern is not observed on the screen after we send many electrons through the double slit apparatus. Instead, the screen shows a pattern like each electron either went through one slit or the other. We will see a picture of this in a sec, and if you are following along the demo, you can try it out for yourself!

Slide 43: But for now, to understand how we currently think about this we have to talk about the act of measuring in quantum mechanics. In quantum, a measurement is probabilistic, which means that if a system is in a superposition state, there's a certain probability associated with getting one of the options in that superposition when you make a measurement on that superposition.

Slide 44: Another way to say this is that the act of making a measurement causes the superposition wave to "collapse" to a single wave. In quantum mechanics, we refer to the state of the system as a "wavefunction", so when we have a wavefunction that is a superposition of two waves, when we make a measurement, that superposition "wavefunction" collapses to just one of the single waves that makes up that superposition.

Slide 45: The chance of the superposition collapsing to one wave or the other depends on the square of the coefficients in the superposition. Then once the wavefunction collapses, it just remains a non-superposition state, with 100% probability of being in that non-superposition state.

Slide 46: Here we can see what happens using the demo. If we look at the high-intensity tab, where we are shining many many electrons through the double slit at a time, not just one at a time, we can see that the interference pattern forms when we just shine those electrons right on through. However, if we put a detector on one of the slits, so that we can tell if the electron goes through slit 1 or slit 2, then because the detector is there, the interference pattern is lost. Instead, it just looks as it would if a non-superposition (electron) wave independently went through slit 1 or slit 2. The detector (i.e., that measurement to determine where the electron is at), collapses the wavefunction so that the electron is not in a superposition of going through slit 1 and slit 2 with equal 50% probability, but instead, is in a wavefunction that either went through slit 1 with 100% probability or went through slit 2 with 100% probability.

Slide 47: Ok, so now that we have seen what happens when we make a quantum measurement on a superposition for real quantum systems, we can explore more how it works

using quantum chess!

Slide 48: For this activity, we will use the Quantum Fork puzzle

Slide 49: In this puzzle, the knight is supposed to capture the rook, so we need to find a way to do that with a superposition.

Slide 50: If the knight moves to B5, then the rook at A7 will move away to avoid capture.

Slide 51: If the knight moves to E2, then the rook at G1 will move away to avoid capture.

Slide 52: So we can create a superposition state for the knight on B5 and E2

Slide 53: Then one rook will try to escape, but we can still try to capture the rook at G1 using the knight at E2

Slide 54: Here is a visual to help you see the individual states that make up the superpositions state. If the superposition collapses to the knight at E2, then capture is successful. If the superposition collapses to the knight at B5, then capture is not successful.

Slide 55: There are two possible outcomes you can get, even if you made the correct quantum move. There is a 50% chance the knight is at E2, and so if it is, then it can successfully capture the rook! There is also a 50% chance the knight is at B5, and if that's the case, then it can't capture the rook. It turns out, this is behavior is representing what happens in quantum mechanics. For a quantum measurement of a superposition state, the outcome will always "collapse" to one of the individual states in the superposition!

Slide 56: Ok, so now you can try it on your own, and we will see the results generated from the group! Let's test to see if we indeed have a 50/50 chance of being successful in the quantum fork activity. Go ahead and run 12 trials of the Quantum Fork puzzle on your own and record the trials using this Google form. Only count outcomes that are correct quantum move. Do not count outcomes that use the wrong move, which would give the output shown here.

Slide 57: Now take a look at the distribution of results we got! Most people got 3 out of 6 successful trials, and some got close to that, and a few got things a little different than that. The average number of successful trials is 3/6, which indicates that It's most likely

that 50% of the trials are successful.

Slide 58: So in summary, today we introduced the definition of a Qubit as a superposition of two states, where the coefficients squared for each of the individual states must square to 100%. Even though the qubit takes into account both states, when a measurement is performed, just one of the states in the superposition will result. The coefficient squared for each individual state is the probability of measuring that particular individual state. So for example, if we have a qubit where the coefficients squared equals $\frac{1}{2}$ for each state, then we have a 50% chance of measuring state zero and 50% chance of measuring state one.

Slide 59: We've covered a lot of basic quantum mechanics principles today, so we won't go into too much more detail, but we will leave you with some thoughts about the potential power of quantum computing! In reality, we can use multiple bits to represent more complicated information. Here is shown how we can use four bits to code the first 16 numbers. So imagine what is possible if each of these bits could be a qubit!

Slide 60: With the emerging feasibility of quantum simulations, which helps predict the properties of new molecules, engineers will be able to consider molecule configurations that would otherwise be challenging to model. RSA cryptography runs on the principle that the product of two large prime numbers can't be factored in our lifetimes. Faster computing speed, including AI-powered algorithms, have long been a source of advantage in financial markets (where hedge funds vie to get millisecond advantages in obtaining price information). Quantum computing has the potential to impact all of these diverse applications in the future, and you can get involved!
